# In situ monolayer patch clamp of acutely stimulated human iPSC-derived cardiomyocytes promotes consistent electrophysiological responses to SK channel inhibition

**DOI:** 10.1038/s41598-024-53571-6

**Published:** 2024-02-07

**Authors:** Andrew S. Butler, Raimondo Ascione, Neil V. Marrion, Stephen C. Harmer, Jules C. Hancox

**Affiliations:** 1 School of Physiology, Pharmacology and Neuroscience, University of Bristol, Bristol, BS8 1TD UK; 2 Bristol Heart Institute and Translational Biomedical Research Centre, Faculty of Health Science, University of Bristol, Bristol, BS2 8HW UK

**Keywords:** Electrophysiology, Heart stem cells, Induced pluripotent stem cells

## Abstract

Human induced pluripotent stem cell-derived cardiomyocytes (iPSC-CMs) represent an in vitro model of cardiac function. Isolated iPSC-CMs, however, exhibit electrophysiological heterogeneity which hinders their utility in the study of certain cardiac currents. In the healthy adult heart, the current mediated by small conductance, calcium-activated potassium (SK) channels (I_SK_) is atrial-selective. Functional expression of I_SK_ within atrial-like iPSC-CMs has not been explored thoroughly. The present study therefore aimed to investigate atrial-like iPSC-CMs as a model system for the study of I_SK_. iPSCs were differentiated using retinoic acid (RA) to produce iPSC-CMs which exhibited an atrial-like phenotype (RA-iPSC-CMs). Only 18% of isolated RA-iPSC-CMs responded to SK channel inhibition by UCL1684 and isolated iPSC-CMs exhibited substantial cell-to-cell electrophysiological heterogeneity. This variability was significantly reduced by patch clamp of RA-iPSC-CMs in situ as a monolayer (iPSC-ML). A novel method of electrical stimulation was developed to facilitate recording from iPSC-MLs via In situ **M**onolayer **P**atch clamp of **A**cutely **S**timulated iPSC-**C**Ms (IMPASC). Using IMPASC, > 95% of iPSC-MLs could be paced at a 1 Hz. In contrast to isolated RA-iPSC-CMs, 100% of RA-iPSC-MLs responded to UCL1684, with APD_50_ being prolonged by 16.0 ± 2.0 ms (p < 0.0001; *n* = 12). These data demonstrate that in conjunction with IMPASC, RA-iPSC-MLs represent an improved model for the study of I_SK_. IMPASC may be of wider value in the study of other ion channels that are inconsistently expressed in isolated iPSC-CMs and in pharmacological studies.

## Introduction

Human induced pluripotent stem cells (iPSCs) represent a potentially unlimited source of cells and their differentiation into iPSC-derived cardiomyocytes (iPSC-CMs) allows large numbers of cells to be generated for experimentation^1,2^. These cells represent a useful model for the study of human cardiac function without being dependent on tissue collection from patients burdened by disease^3–6^. Differentiation of iPSC-CMs using conventional protocols produces a population of cells with a predominantly ventricular-like phenotype, but the presence of retinoic acid (RA) during the mesodermal stage of differentiation produces iPSC-CMs which exhibit a more atrial-like phenotype^7–10^. iPSC-CMs have shown value as a drug screening platform, an in vitro model of cardiac disease, and as a model system for increased understanding of basic cardiac function^11–15^.

Despite their utility, iPSC-CMs are limited to some degree by an ‘immature’ phenotype. Isolated iPSC-CMs typically exhibit a rounded shape in comparison with the rod-shaped structure of an adult cardiomyocyte (CM) and also demonstrate a comparatively depolarised membrane potential and significantly slower action potential (AP) upstroke velocity^16–18^. Isolated iPSC-CMs also exhibit significant cell-to-cell electrophysiological variability. Functional expression of some currents, including the rapidly activating delayed rectifier K^+^ current (I_Kr_), the current mediated by the Na^+^/Ca^2+^ exchanger (I_NCX_), and the fast Na^+^ current (I_Na_), appears to be consistent amongst isolated iPSC-CMs^19–22^. However, only a proportion of ventricular-like iPSC-CMs have been shown to respond to blockade of other ionic currents including the slow delayed rectifier K^+^ current (I_Ks_), the inwardly rectifying potassium current (I_K1_) and the L-type calcium current (I_Ca,L_)^20,22–24^. Similarly, only a proportion of atrial-like iPSC-CMs respond to inhibition of the atrial-selective ultrarapid outward potassium current (I_Kur_; ~ 60%), whilst other atrial-selective currents, including that mediated by small conductance, calcium-activated potassium (SK) channels (I_SK_) have not been well characterised^25^.

The use of multicellular iPSC-CM preparations such as two-dimensional (2D) monolayers (iPSC-MLs) or three-dimensional (3D) ‘engineered heart tissues’ (EHTs) instead of isolated iPSC-CMs may reduce experimental variability. In such preparations, the preservation of electrical coupling between cells means that electrophysiological recordings reflect the summation of a wider pool of currents which influence the measured membrane potential^26,27^. Additionally, experiments on isolated iPSC-CMs typically involve prolonged periods of culture as single cells^10,22,27^. This is known to promote electrophysiological remodelling in isolated adult CMs and may therefore contribute to the heterogeneity exhibited by isolated iPSC-CMs^28–30^.

At present, intracellular recording of electrophysiological data from 2D iPSC-MLs predominantly depends upon techniques that are unable to simultaneously stimulate AP firing at a constant cycle length, hindering the interpretation of drug-induced changes in AP duration (APD). Alternative methods rely upon voltage mapping techniques such as optical mapping or the use of multi-electrode arrays^31–33^. Whilst these exhibit some advantages over intracellular recordings, they are limited by their inability to quantify voltage and by the resolution of response. The generation of 3D EHTs is experimentally challenging for many laboratories due to the requirement for a large cell number, the scalability of cell culture processes and the purchase/fabrication of specialised culture platforms^19,34–36^. Taken together, these factors highlight that the development of a simple 2D system in which iPSC-MLs can be stimulated whilst intracellular recordings are made concurrently would have utility.

The initial motivation for this study was to characterise the functional expression of SK channels in iPSC-CMs. To this end, we differentiated iPSCs (± RA) to produce iPSC-CMs with an atrial- or ventricular-like phenotype and analysed SK channel activity. We show that although RA induces an atrial-like phenotype, only a sub-population (< 20%) of isolated RA-iPSC-CMs respond to SK channel inhibition and that isolated iPSC-CMs exhibit significant cell-to-cell variability.

In an effort to bypass the inherent cell-to-cell variability in ion channel activity recorded in isolated iPSC-CMs we developed a novel method for In situ **M**onolayer **P**atch clamp of **A**cutely** S**timulated iPSC-**C**Ms (IMPASC), which allowed concurrent whole-cell patch clamp recordings of APs to be made at a constant cycle length. When recording from RA-iPSC-MLs using the IMPASC method, 100% of monolayers responded to SK channel inhibition and electrophysiological heterogeneity, as compared to isolated iPSC-CMs, was significantly reduced. In summary, the present study highlights that SK channels are functionally expressed in atrial-like iPSC-CMs and that the novel IMPASC method may have wider benefit in the study of other ion channels that are inconsistently expressed in isolated iPSC-CMs.

## Results

### Isolated iPSC-CMs exhibit inconsistent responses to modulation of I_SK_ and I_K,ACh_

**Figure 1:**
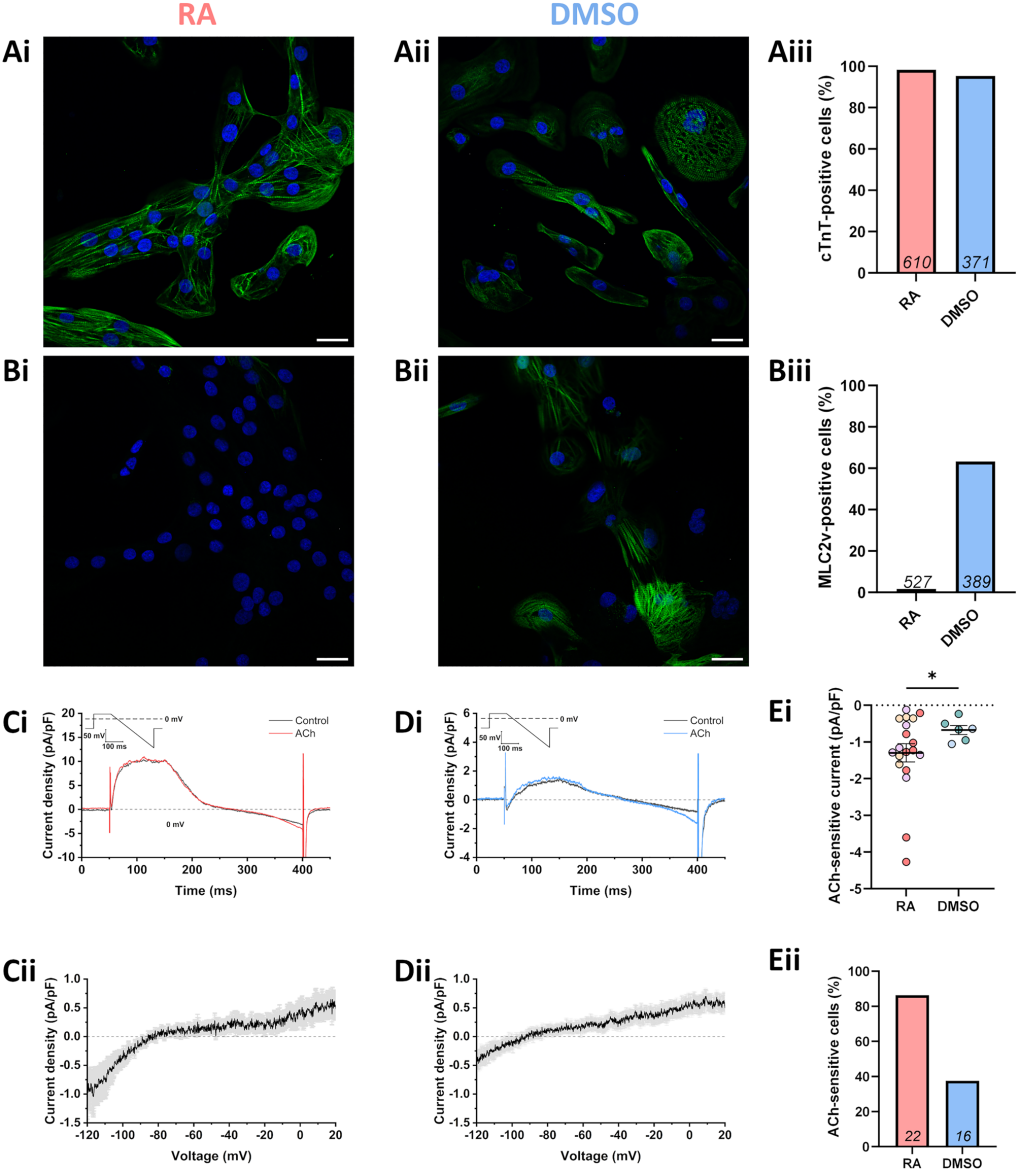
RA-iPSC-CMs exhibit a more atrial-like phenotype than DMSO-iPSC-CMs. (**A**) Representative images of RA-iPSC-CMs (**Ai**) and DMSO-iPSC-CMs (**Aii**) immunostained for cTnT (green). Scale bars represent 25 µm and cell nuclei (DAPI) are shown in blue. (**Aiii**) Percentage of cells positive for cTnT, as calculated from at least three images of at least two independent batches of cells. The number of cells analysed are shown within bars. (**B**) Representative images of RA-iPSC-CMs (**Bi**) and DMSO-iPSC-CMs (**Bii**) immunostained for MLC2v (green). Scale bars represent 25 μm and cell nuclei (DAPI) are shown in blue. (**Biii**) Percentage of cells positive for MLC2v. The number of cells analysed are shown within bars. Data collected from three independent differentiation batches. (**C**, **D**) Representative currents from ACh-sensitive RA-iPSC-CMs (**Ci**) and DMSO-iPSC-CMs (**Di**) under control conditions (black) and following application of 1 μM ACh (coloured) using the protocol inset. Mean ACh-sensitive current (± SEM) from cells which responded to drug, where mean is derived from *n* = 19 (RA-iPSC-CMs; **Cii**) and *n* = 5 (DMSO-iPSC-CMs; **Dii**). (**Ei**) Amplitude of current elicited by application of ACh, measured at − 120 mV. Different shades represent the three independent differentiation batches. Data points are only shown for the cells which responded to ACh. Statistics represent an unpaired Student’s t-test. (**Eii**) Percentage of isolated iPSC-CMs which responded to application of ACh. Total number of cells from which recordings were made in each group are shown within bars. Currents recorded from three independent differentiation batches of RA-iPSC-CMs and two batches of DMSO-iPSC-CMs.

The production of a pure population of iPSC-CMs was confirmed by immunostaining for the cardiac isoform of troponin T (cTnT). Over 95% of RA-iPSC-CMs and DMSO-iPSC-CMs were positive for cTnT, demonstrating the majority of cells to be cardiomyocytes (Fig. 1A). The development of an atrial-like phenotype in RA-iPSC-CMs was indicated by the absence of the ventricular marker MLC2v (Fig. 1Bi,Biii), whilst the majority of DMSO-iPSC-CMs did express MLC2v (Fig. 1Bii,Biii) as has been previously established^8,9^. In agreement with previous work, differentiation using RA also increased the incidence (19/22 RA-iPSC-CMs vs. 5/16 DMSO-iPSC-CMs) and amplitude (-1.30 ± 0.25 vs. -0.67 ± 0.12 pA/pF; p < 0.05; measured at -120 mV; Fig. 1C–E) of the acetylcholine (ACh)-activated potassium current (I_K,ACh_; activated by 1 µM ACh), supporting the development of a more atrial-like phenotype in RA-iPSC-CMs^8,9^.

**Figure 2:**
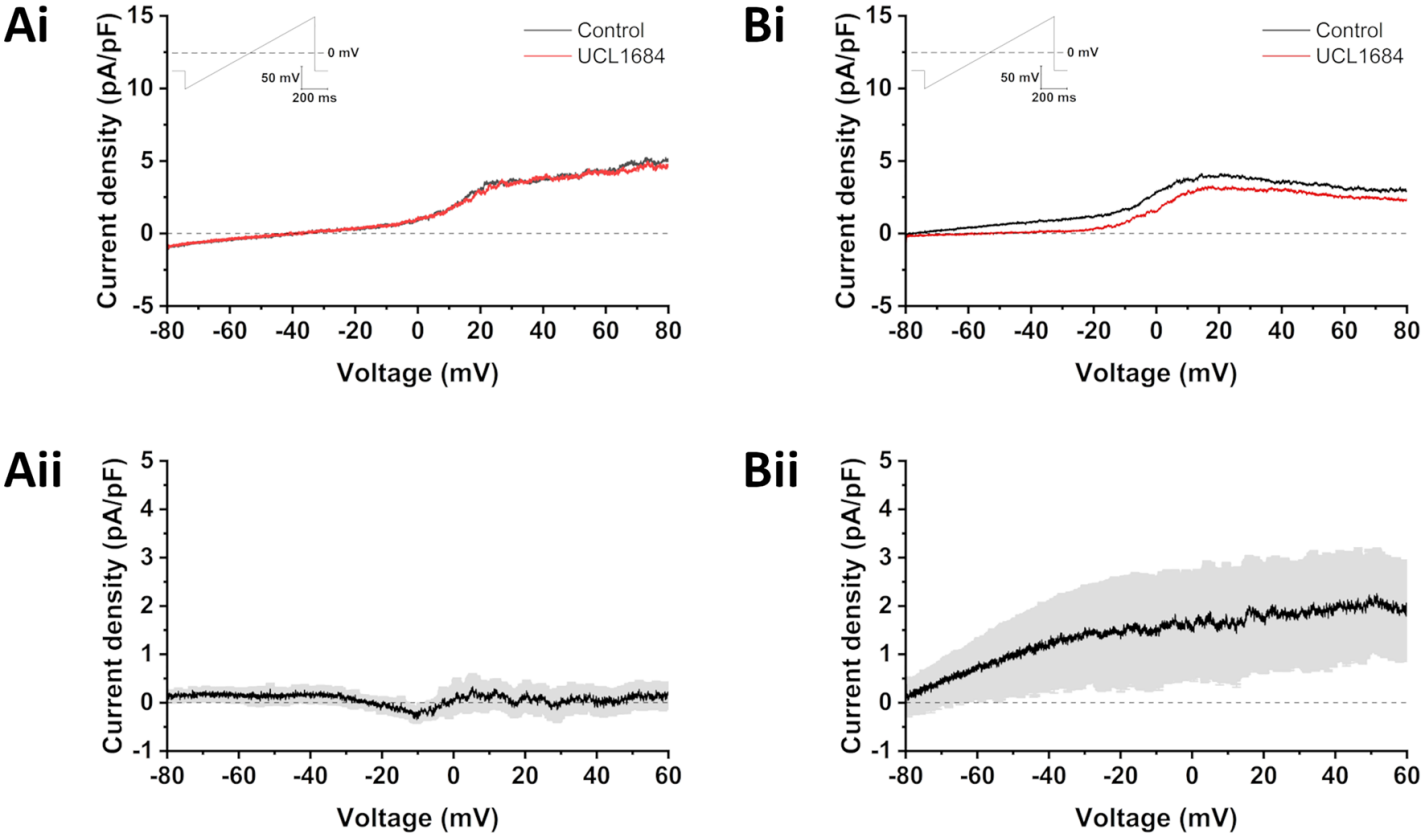
UCL1684 sensitivity in isolated RA-iPSC-CMs. Representative traces from isolated RA-iPSC-CMs which were unresponsive (**Ai**; *n* = 14/17) or responsive (**Bi**; *n* = 3/17) to application of 100 nM UCL1684. Currents were elicited by the protocol inset. Mean (± SEM) UCL1684-sensitive current in isolated RA-iPSC-CMs in which I_SK_ was not (**Aii**; *n* = 14) or was (**Bii**;* n* = 3) recorded following application of 100 nM UCL1684. Data were pooled from three independent differentiation batches of RA-iPSC-CMs.

Within the heart, I_SK_ is an atrial-selective current presently under investigation as a novel target for the treatment of atrial fibrillation^37–41^, but functional expression of I_SK_ in isolated RA-iPSC-CMs has not been thoroughly investigated to the best of our knowledge. Whole-cell patch clamp was performed using an internal solution containing 500 nM free Ca^2+^ in order to fully activate I_SK_. 14/17 cells did not respond to application of 100 nM UCL1684 (Fig. 2A), whereas the remaining 3 cells did exhibit UCL1684-sensitive current (1.20 ± 0.66 pA/pF; measured at − 20 mV; Fig. 2B). Taken together, these data demonstrate that whilst RA-iPSC-CMs exhibit a more atrial-like phenotype than DMSO-iPSC-CMs, a heterogenous population exists in which responses to activation of I_K,ACh_ are inconsistent and I_SK_ is absent in > 80% of isolated cells.

### Monolayers of iPSC-CMs exhibit reduced heterogeneity of action potential duration

Patch clamp recording of isolated iPSC-CMs requires enzymatic digestion and a period of time spent in culture as single cells. It is possible that these experimental conditions may contribute to the heterogeneity recorded in the data presented above, given that significant ion channel remodelling occurs in adult CMs following dissociation and prolonged time in culture^42^. This is perhaps best exemplified by I_K1_, which can decline by > 90% following culture of isolated CMs for < 1 week^28,43^. As an alternative to cell dissociation, RA-iPSC-CMs were instead plated as monolayers (RA-iPSC-MLs) two weeks prior to experimentation. Previous work has shown that prolonging time in culture prior to dissociation increases cellular maturity^44,45^, and so experiments were also performed in which RA-iPSC-CMs were cultured for ~ 30 or ~ 60 days prior to dissociation into single cells and then patched at day 35 ± 5 (isolated D35) or at day 65 ± 5 (isolated D65; see methods for more details). As voltage clamp experiments cannot be performed on RA-iPSC-MLs due to the inability to clamp the membrane potential of a spontaneously active syncytium, voltage clamp measurements from monolayers were not attempted and, instead, spontaneous APs were recorded to compare groups.

**Figure 3:**
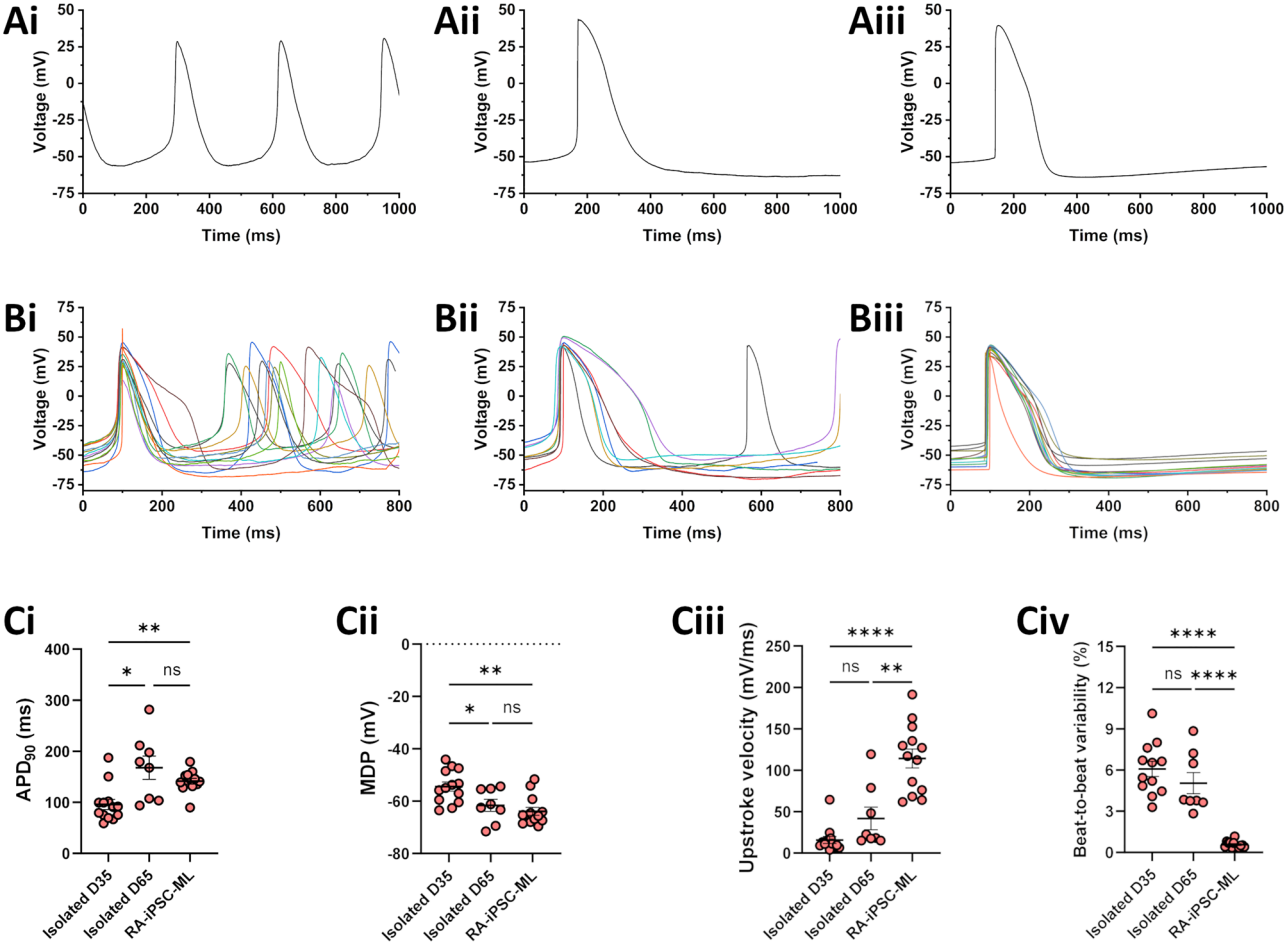
Spontaneous APs in isolated RA-iPSC-CMs and monolayers (RA-iPSC-MLs). (**A**) Representative recording of spontaneous APs recorded from isolated D35 RA-iPSC-CMs (**Ai**), isolated D65 RA-iPSC-CMs (**Aii**) or RA-iPSC-MLs (**Aiii**). (**B**) Overlay of all spontaneous APs recorded from isolated D35 RA-iPSC-CMs (**Bi**), isolated D65 RA-iPSC-CMs (**Bii**) or RA-iPSC-MLs (**Biii**) used for data analysis. (**C**) APD_90_ (**Ci**), maximum diastolic potential (**Cii**), maximum upstroke velocity (**Ciii**) and beat-to-beat variability in APD_90_ (**Civ**) recorded from each group. Data are presented as mean ± SEM. All action potentials recorded using whole-cell current clamp at 37 °C. Statistics represent Brown-Forsythe ANOVA test. All data recorded from one batch of RA-iPSC-CMs.

Spontaneous APs from isolated D35 iPSC-CMs, isolated D65 iPSC-CMs and RA-iPSC-MLs are shown in Fig. 3A,B. Extending culture time prolonged APD_90_ in isolated cells from 95.9 ± 10.0 ms (D35; *n* = 13) to 167.7 ± 22.8 ms (D65; *n* = 8; p < 0.05; Fig. 3Ci) and produced cells with a more hyperpolarised maximum diastolic potential (MDP; − 54.4 ± 1.7 vs. − 61.6 ± 2.3 mV; p < 0.05; Fig. 3Cii). A trend towards an increased maximum upstroke velocity was also recorded (15.8 ± 4.3 vs. 41.8 ± 13.6 mV/ms; p > 0.05; Fig. 3Ciii). Taken together, this suggests that isolated D65 RA-iPSC-CMs exhibit a more mature electrophysiological phenotype than isolated D35 RA-iPSC-CMs, which agrees well with previous work^20,44,46^.

RA-iPSC-MLs (Fig. 3Aiii,Biii) exhibited a similar APD_90_ (141.5 ± 5.7 ms; p > 0.05; *n* = 13; Fig. 3Ci) and MDP (− 63.9 ± 1.5 mV; p > 0.05; Fig. 3Cii) to isolated D65 RA-iPSC-CMs. The maximum upstroke velocity recorded from RA-iPSC-MLs (114.2 ± 11.4 mV/ms) was significantly faster than that recorded in either isolated D35 or D65 cells (p < 0.0001 & p < 0.01 respectively; Fig. 3Ciii). Although the mean APD_90_ (as well as APD_30_ and APD_50_; see Fig. S1) was similar between isolated D65 RA-iPSC-CMs and RA-iPSC-MLs, the monolayers exhibited significantly reduced variability at APD_30_, APD_50_ (p < 0.05 & 0.01 respectively; F-test; Fig. S1) and APD_90_ (p < 0.001; F-test; Fig. 3Ci). This reduction in heterogeneity is evident when spontaneous action potential recordings are aligned (Fig. 3B). In addition to the reduced inter-cellular heterogeneity, a significant reduction in beat-to-beat variability of APD_90_ was exhibited by RA-iPSC-MLs (Fig. 3Civ). Isolated D35 and D65 RA-iPSC-CMs showed similar variability (6.08 ± 0.55% and 5.05 ± 0.77% respectively; p > 0.05), whilst RA-iPSC-MLs exhibit beat-to-beat variability of only 0.57 ± 0.07% (p < 0.0001 vs. isolated D35 and D65).

### Development of IMPASC: a new method for simultaneous stimulation and whole-cell electrophysiological recording from iPSC-MLs

The reduced electrophysiological heterogeneity recorded in iPSC-MLs raised the possibility that more consistent responses to ion channel modulation may be recorded from monolayers than are recorded from isolated cells. Drug-induced alterations in APD (and/or membrane potential) are expected to perturb AP firing rate and changes to rate could influence APD independent of drug actions. Consequently, in order to accurately measure drug-induced changes in ion channel activity from iPSC-MLs, it is important to record APs elicited at user-defined stimulation rates rather than merely study the effects on spontaneous activity. To the best of our knowledge, a method for patch clamp recordings of triggered APs in 2D monolayer preparations of iPSC-CMs has not hitherto been established.

**Figure 4:**
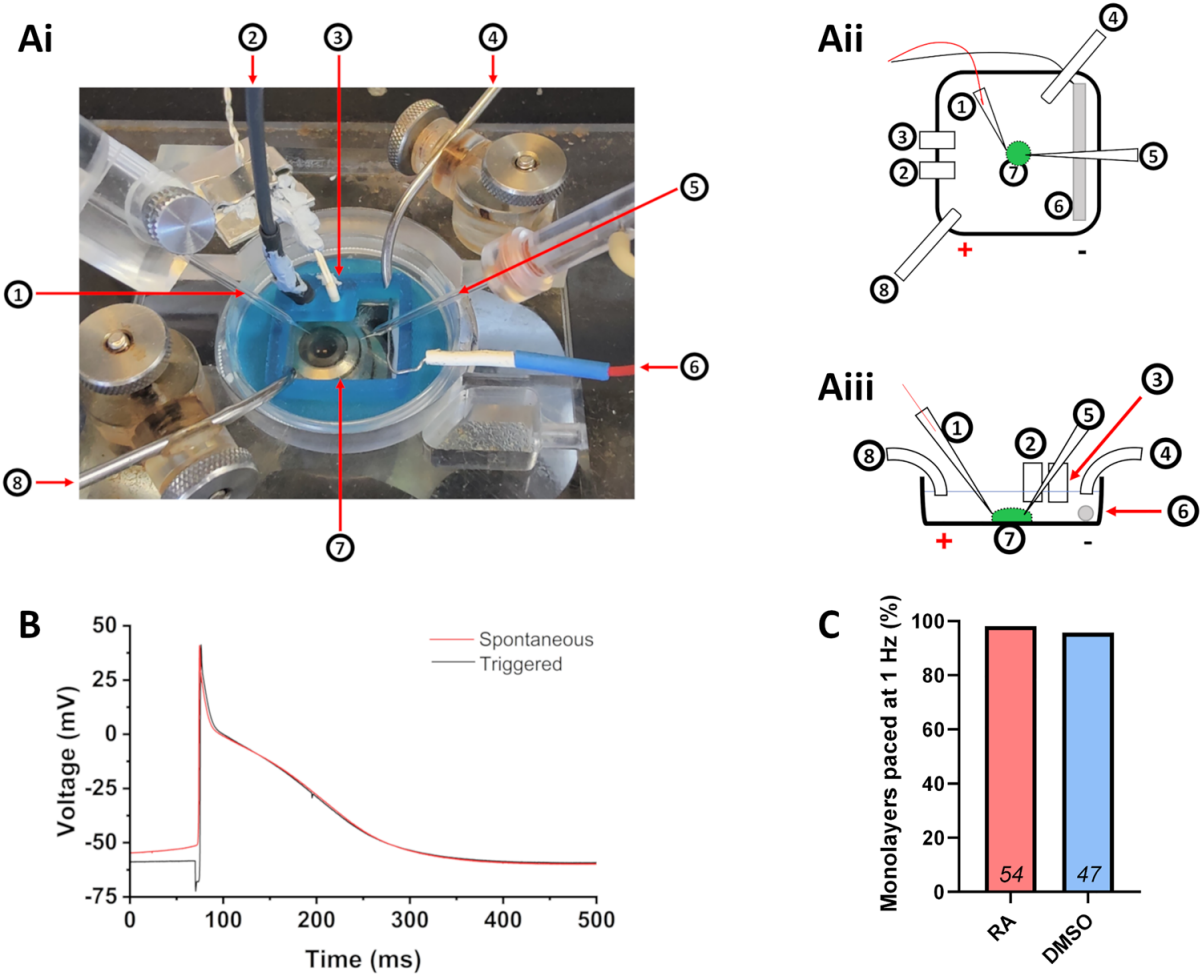
Using IMPASC to stimulate monolayers and record action potentials. (**A**) Photo (**Ai**) and schematics (**Aii**, **Aiii**) of the bath organisation for IMPASC (In situ Monolayer Patch clamp of Acutely Stimulated iPSC-CMs). Numbers correspond to the: stimulating electrode (1), ground electrode (2; for patch electrode), temperature probe (3), outflow tube (4), patch electrode (5), platinum ground electrode (6; for simulating electrode), chamber for coverslip of cells (7; iPSC-ML represented by green circle) and inflow tube (8). (**B**) Whole-cell current clamp recording of a spontaneous (red) or triggered (black) action potential recorded from an RA-iPSC-ML. Stimulation threshold was confirmed by visual check of the monolayer for contraction and a stimulation of threshold + 20% was applied. Action potentials recorded using whole-cell current clamp at 37 °C. (**C**) Percentage of iPSC-MLs which could be triggered at a rate of 1 Hz using the methodology detailed. The total number of iPSC-MLs from which recordings were made in each group are shown within bars. Data pooled from three independent differentiation batches of RA-iPSC-MLs and DMSO-iPSC-MLs.

Initial attempts at monolayer stimulation by current injection via the patch pipette (as used in studies of isolated adult CMs^6,28^) or by traditional field stimulation (as used to stimulate multicellular preparations^9,35^) caused large stimulation artefacts which prevented APs from being accurately recorded (Fig. S2). To improve the suitability of stimulation methods, a platinum electrode (stimulating electrode) was placed inside a glass patch pipette filled with external solution and a second platinum electrode was placed in the recording chamber to serve as a ground electrode (Fig. 4A). The stimulating electrode was mounted on a micromanipulator to allow positioning in close proximity to the monolayer of iPSC-CMs (Fig. 4A). These electrodes were connected to an isolated constant current stimulator (DS3, Digitimer) which could then be triggered by commands delivered from the recording software. Injection of a 200–700 µA hyperpolarising current triggered AP firing and contraction of iPSC-MLs. In situ **M**onolayer **P**atch clamp of **A**cutely **S**timulated iPSC-**C**Ms (IMPASC) enabled APs to be recorded at 1 Hz in > 95% of iPSC-MLs (98.2% of 54 RA-iPSC-MLs and 95.8% of 47 DMSO-iPSC-MLs; Fig. 4C) with only a small stimulation artefact present (Fig. 4B).

### Stimulated action potential properties of iPSC-MLs

**Figure 5:**
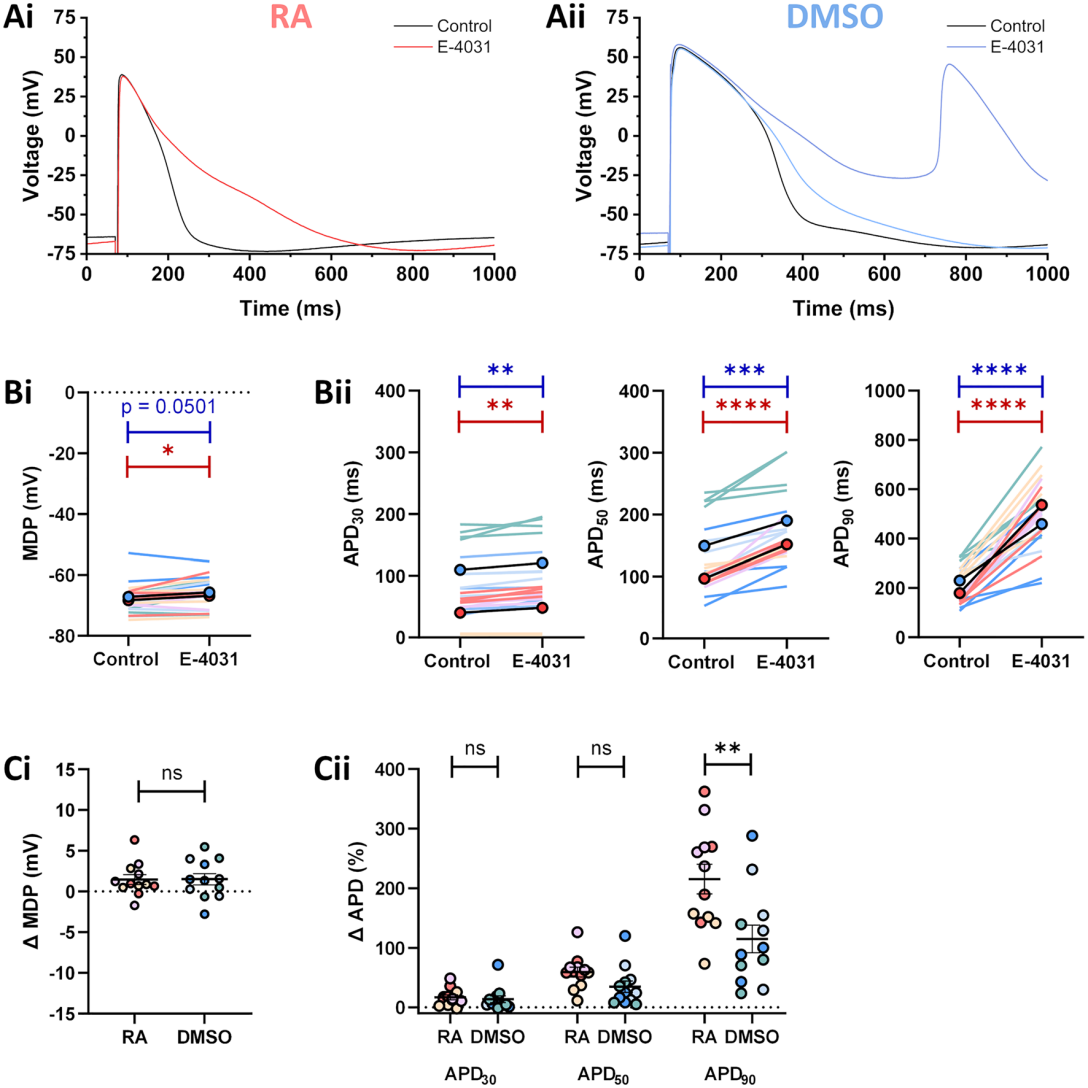
Effects of 1 µM E-4031 on iPSC-MLs. (**A**) Representative traces from RA-iPSC-MLs (**Ai**) and DMSO-iPSC-MLs (**Aii**) paced at 1 Hz under control conditions (black) and following application of 1 μM E-4031 (red/blue). Where multiple traces are shown for E-4031, the lighter shade represents the final sweep before abnormal depolarisations occurred. (**B**) Effect of 1 μM E-4031 on MDP (**Bi**) and APD (**Bii**) in RA-iPSC-MLs (reds) and DMSO-iPSC-MLs (blues). The circles with black lines represent the mean of all batches. Statistics represent paired Student’s t-tests comparing APD before and after drug application. (**C**) Change in MDP (**Ci**) and APD (**Cii**) in iPSC-MLs following application of 1 μM E-4031. Data are presented as mean ± SEM. Statistics represent unpaired Student’s t-tests comparing the effects on RA- and DMSO-iPSC-MLs. For all figures, different shades represent the three independent differentiation batches. Batches were pooled for comparisons between RA and DMSO. When E-4031 triggered EADs, the final sweeps before EADs occurred were used to calculate APD. *n* = 12 for both groups, consisting of 4 from each differentiation batch. All recordings made at 37 °C.

**Figure 6:**
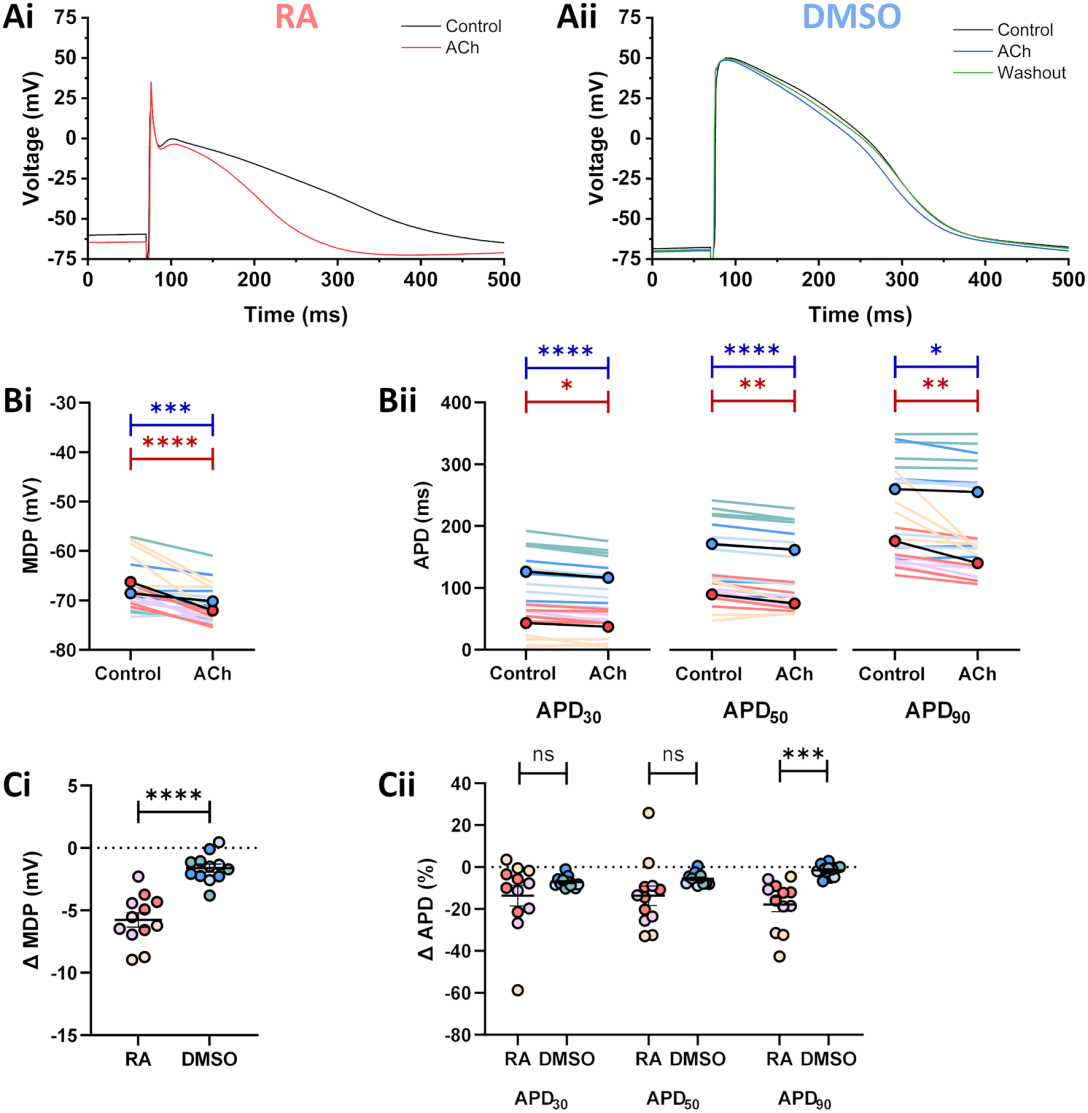
Effects of 1 µM ACh on iPSC-MLs. (**A**) Representative traces from RA-iPSC-MLs (**Ai**) and DMSO-iPSC-MLs (**Aii**) paced at 1 Hz under control conditions (black) and following application of 1 μM ACh (red/blue). (**B**) Effect of 1 μM ACh on MDP (**Bi**) and APD (**Bii**) in RA-iPSC-MLs (reds) and DMSO-iPSC-MLs (blues). The circles with black lines represent the mean of all batches. Statistics represent paired Student’s t-tests comparing APD before and after drug application. (**C**) Change in MDP (**Ci**) and APD (**Cii**) in iPSC-MLs following application of 1 μM ACh. Data are presented as mean ± SEM. Statistics represent unpaired Student’s t-tests comparing the effects on RA- and DMSO-iPSC-MLs. For all figures, different shades represent the three independent differentiation batches. Batches were pooled for comparisons between RA and DMSO. *n* = 12 for both groups, consisting of 4 from each differentiation batch. All recordings made at 37 °C.

**Figure 7:**
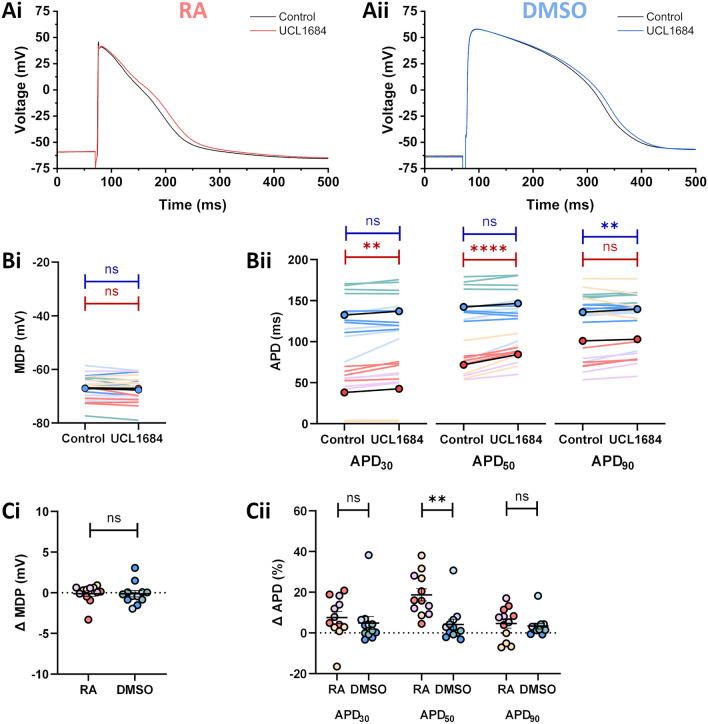
Effects of 100 nM UCL1684 on iPSC-MLs. (**A**) Representative traces from RA-iPSC-MLs (**Ai**) and DMSO-iPSC-MLs (**Aii**) paced at 1 Hz under control conditions (black) and following application of 100 nM UCL1684 (red/blue). (**B**) Effect of 100 nM UCL1684 on MDP (**Bi**) and APD (**Bii**) in RA-iPSC-MLs (reds) and DMSO-iPSC-MLs (blues). The circles with black lines represent the mean of all batches. Statistics represent paired Student’s t-tests comparing APD before and after drug application. (**C**) Change in MDP (**Ci**) and APD (**Cii**) in iPSC-MLs following application of 100 nM UCL1684. Data are presented as mean ± SEM. Statistics represent unpaired Student’s t-tests comparing the effects on RA- and DMSO-iPSC-MLs. For all figures, different shades represent the three independent differentiation batches. Batches were pooled for comparisons between RA and DMSO. *n* = 12 for both groups, consisting of 4 from each differentiation batch. All recordings made at 37 °C.

IMPASC was used to characterise differences in AP morphology between RA- and DMSO-iPSC-MLs paced at a constant cycle length of 1000 ms. Data were collected from 3 independent differentiation batches to ensure reproducibility. DMSO-iPSC-MLs from each batch exhibited broadly similar AP properties, with a clear plateau phase present following depolarisation (Fig. S3B; also see representative traces in Figs. 5, 6 and 7Aii). RA-iPSC-MLs exhibited two different AP morphologies: one with rapid phase one repolarisation followed by a relative plateau phase at ~ 0 mV; and one consisting of short, triangular APs (Fig. S3A; also see representative traces in Figs. 5, 6 and 7Ai). Data from the three independent batches were pooled for analysis.

Paced RA-iPSC-MLs exhibited a less negative MDP than did DMSO-iPSC-MLs (− 64.8 ± 0.7 [RA; *n* = 53] vs. − 67.4 ± 0.7 mV [DMSO; *n* = 45]). APD was shorter in RA-iPSC-MLs at APD_30_ (40.5 ± 4.0 [RA] vs. 128.8 ± 4.6 ms [DMSO]), APD_50_ (97.8 ± 4.2 [RA] vs. 173.2 ± 5.7 ms [DMSO]) and APD_90_ (220.8 ± 13.3 [RA] vs. 283.4 ± 10.2 ms [DMSO]). RA-iPSC-MLs also exhibited a lower AP amplitude and plateau amplitude (101.6 ± 1.6 mV and 82.2 ± 2.8 mV respectively) than DMSO-iPSC-MLs (113.0 ± 2.1 mV and 110.0 ± 2.2 mV). The plateau fraction (APD_30_/APD_90_) was reduced in RA-iPSC-MLs (0.241 ± 0.025) as compared with DMSO-iPSC-MLs (0.459 ± 0.009). These properties are summarised in Table 1 and individual data points are shown in Fig. S3C. For comparison, spontaneous AP properties are shown in Fig. S4 and Table S1. The reduced APD, plateau and AP amplitude, and plateau fraction of RA-iPSC-MLs in comparison with DMSO-iPSC-MLs are characteristic of a more atrial-like phenotype, supporting the data shown in Fig. 1.

**Table 1:** Properties of action potentials in iPSC-MLs paced at 1 Hz.

Property	RA (*n* = 53)	DMSO (*n* = 45)	Significance
MDP (mV)	− 64.8 ± 0.7	− 67.4 ± 0.7	p < 0.01
APD_30_ (ms)	40.5 ± 4.0	128.8 ± 4.6	p < 0.0001
APD_50_ (ms)	97.8 ± 4.2	173.2 ± 5.7	p < 0.0001
APD_90_ (ms)	220.8 ± 13.3	283.4 ± 10.2	p < 0.001
AP amplitude (mV)	101.6 ± 1.6	113.0 ± 2.1	p < 0.0001
Plateau amplitude (mV)	82.2 ± 2.8	110.0 ± 2.2	p < 0.0001
Plateau fraction (APD_30_/APD_90_)	0.241 ± 0.025	0.459 ± 0.009	p < 0.0001

Comparison of properties recorded from APs stimulated at 1 Hz in RA-iPSC-MLs and DMSO-iPSC-MLs. Statistics represent unpaired Student’s t-test, with Welch’s correction where appropriate (APD_50_, plateau amplitude and APD_30_/APD_90_). Data are presented as mean ± SEM. All recordings made at 37 °C. Data pooled from three independent differentiation batches of RA-iPSC-MLs and DMSO-iPSC-MLs. Individual data points can be seen in Fig. S3C.

### Effects of E-4031 on iPSC-MLs

To further investigate the effectiveness of the IMPASC technique, the effects of the I_Kr_ inhibitor E-4031 on AP properties were investigated. Repolarisation of iPSC-CMs and EHTs has previously been shown to be critically dependent upon I_Kr_, and its inhibition may subsequently trigger early afterdepolarisations (EADs)^20,35^. Representative traces of RA- and DMSO-iPSC-MLs in the absence and presence of 1 µM E-4031 are shown in Fig. 5A. The MDP was depolarised by 1.5 ± 0.6 mV and 1.5 ± 0.7 mV in RA-iPSC-MLs and DMSO-iPSC-MLs respectively following application of E-4031 (Fig. 5Bi,Ci). Application of E-4031 significantly prolonged repolarisation at APD_30_, APD_50_ and APD_90_ in both RA- and DMSO-iPSC-MLs (Fig. 5Bii,Cii; *n* = 12 for both groups) but augmentation was larger during late repolarisation (+ 355.9 ± 28.2 ms [RA] & + 227.9 ± 34.7 ms [DMSO] prolongation at APD_90_) than during early repolarisation (+ 8.1 ± 2.2 ms [RA] & + 11.0 ± 3.4 ms [DMSO] prolongation at APD_30_). EADs were triggered in a lower proportion of RA-iPSC-MLs (6/12) than DMSO-iPSC-MLs (9/12) and the duration of each pro-arrhythmic event was typically longer in DMSO-iPSC-MLs than RA-iPSC-MLs (Fig. S5). 100% of RA- and DMSO-iPSC-MLs responded to application of E-4031.

### Effects of ACh on iPSC-MLs

In the adult heart, I_K,ACh_ is considered to be an atrial selective current^47,48^ and the data shown in Fig. 1 demonstrated that isolated RA-iPSC-CMs exhibited an increased incidence and amplitude of I_K,ACh_ compared with DMSO-iPSC-CMs. Representative traces of RA- and DMSO-iPSC-MLs in the absence and presence of 1 µM ACh are shown in Fig. 6A. Application of ACh hyperpolarised the membrane potential and abbreviated repolarisation in both RA- and DMSO-iPSC-MLs (Fig. 6; *n* = 12 for both groups). ACh hyperpolarised the MDP significantly more in RA-iPSC-MLs (5.8 ± 0.7 mV) than in DMSO-iPSC-MLs (1.6 ± 0.3 mV; p < 0.0001; Fig. 6Bi,Ci). APD_30_ and APD_50_ were shortened in RA-iPSC-MLs and DMSO-iPSC-MLs to a similar degree (6.3 ± 1.7 ms & 14.8 ± 4.1 ms [RA] vs. 9.5 ± 1.4 ms & 9.8 ± 1.5 ms [DMSO]; p > 0.05 for both). RA-iPSC-MLs exhibited increased shortening of APD_90_ compared with DMSO-iPSC-MLs, with ACh application reducing APD_90_ by 35.6 ± 10.2 ms (vs. 4.7 ± 2.1 ms in DMSO; p < 0.01; Fig. 6Bii,Cii). 100% of RA-iPSC-MLs and 83% of DMSO-iPSC-MLs exhibited APD shortening and hyperpolarisation of the diastolic membrane potential in response to application of ACh.

### Effects of SK channel inhibition on iPSC-MLs

The atrial-selective current, I_SK_, has not been well characterised in iPSC-CMs and in Fig. 2 it was demonstrated that < 20% of isolated RA-iPSC-CMs responded to inhibition by 100 nM UCL1684. Representative traces of RA- and DMSO-iPSC-MLs in the absence and presence of 100 nM UCL1684 are shown in Fig. 7A. UCL1684 did not affect the MDP in either group of MLs (− 0.1 ± 0.3 mV [RA] & − 0.1 ± 0.4 mV [DMSO]; Fig. 7Bi,Ci; *n* = 12 for both groups). APD_30_ and APD_50_ were not significantly prolonged by UCL1684 in DMSO-iPSC-MLs (4.5 ± 2.5 ms and 5.4 ± 2.8 ms respectively; p > 0.05 vs. control for both) but prolongation of both of these APD parameters was recorded in RA-iPSC-MLs (4.5 ± 1.3 ms; p < 0.01 vs. control for APD_30_ & 16.0 ± 2.0 ms; p < 0.0001 vs. control for APD_50_; Fig. 7Bii). Prolongation of APD_50_ by application of UCL1684 in RA-iPSC-MLs was therefore significantly greater than that recorded in DMSO-iPSC-MLs (p < 0.01; Fig. 7Cii).

APD_90_ was prolonged by 7.3 ± 2.2 ms (3.3 ± 1.4%; p < 0.01 vs. control) in DMSO-iPSC-MLs but there was no mean change in APD_90_ in RA-iPSC-MLs following application of UCL1684 (+ 4.0 ± 4.4 ms; p > 0.05 vs. control; Fig. 7Bii). This occurred because *prolongation* of APD_90_ was recorded in the differentiation batches which exhibited short, triangular APs (13.2 ± 2.0 ms, 9.3 ± 4.3%; *n* = 8; Fig. S6Ai) but an *abbreviation* of repolarisation was recorded in the differentiation batches which exhibited rapid phase one repolarisation (− 14.4 ± 4.7 ms, − 4.8 ± 1.6%; *n* = 4; Fig. S6Aii). As an abbreviation of repolarisation caused by SK channel inhibition has not previously been reported, experiments were repeated using a second SK channel inhibitor, apamin. Application of 100 nM apamin produced data which mirrored the application of UCL1684 (Fig. S6). In contrast to isolated RA-iPSC-CMs, 100% of RA-iPSC-MLs responded to SK channel inhibition.

## Discussion

### IMPASC reduces electrophysiological heterogeneity

The data presented in Figs. 1, 2 and 3 demonstrate that significant cell-to-cell variability occurs in isolated iPSC-CMs. Pharmacologically, this was highlighted by inconsistent responses to modulation of I_K,ACh_ and I_SK_. Similar data have previously been shown in the study of other cardiac currents: whole-cell patch clamp of isolated iPSC-CMs has previously demonstrated that only ~ 30% of cells isolated at day ~ 30 exhibit recordable I_Ks_^22,23^. In these studies, cells were cultured as single cells for 4–20 days prior to experimentation and so are comparable to the data in the present study. I_Kur_ was absent in ~ 40% of isolated RA-iPSC-CMs which were cultured and patched on similar timelines^25^. 12% (out of 289) of iPSC-CMs isolated from monolayer cultures were shown to lack I_Ca,L_^24^. Similarly, I_K1_ has been reported as only recordable in ~ 50% of isolated iPSC-CMs and hESC-CMs, although the extent to which this is due to technical challenges during recording is unclear^20,49,50^.

In agreement with previous work, prolongation of time in culture led to a more mature electrophysiological phenotype^20,44,46^. However, significant heterogeneity was still prevalent in isolated RA-iPSC-CMs (Fig. 3). APD variability has previously been shown to be reduced in EHTs as compared with isolated iPSC-CMs^50,51^ and this agrees well with our data showing that the 2D monolayer recordings exhibit reduced variability. Although substantial heterogeneity also occurs in isolated adult CMs^52^, this may be exacerbated in iPSC-CMs by periods of culture as isolated cells (2–12 days in the present study) prior to experimentation, which promotes significant ion channel remodelling in adult CMs^28,43^. This period of recovery is necessary due to the fragility of iPSC-CMs following dissociation, however when adult CMs are isolated and placed in culture ion channel downregulation readily occurs. For example, I_K1_ density decreases by > 90% in ~ 6 days and I_Ca,L_ density decreases by ~ 60% in ~ 5 days^28,53,54^. Although the mechanisms which cause remodelling in isolated cardiomyocytes have not been fully elucidated, factors including a loss of cell-to-cell signalling, lack of action potential stimulation and disruption of physical stretching of the CMs all likely contribute^55–59^, and changes in these cues are similarly applicable to iPSC-CMs dissociated from a monolayer. In support of this hypothesis, iPSC-CMs have been shown to undergo rapid structural remodelling following dissociation^60^. In adult CMs, SK channels co-localise with L-type calcium channels and so structural remodelling combined with a reduction in I_Ca,L_ may be associated with a reduction in I_SK_ and contribute to the limited response to UCL1684 shown in RA-iPSC-CMs in Fig. 2^61,62^. The use of multicellular preparations in the study of iPSC-CMs prevents any opportunity for such remodelling to occur.

Retention of a syncytium also co-ordinates electrical activity between cells and supresses cell-to-cell electrical variability, and so patch clamp of cells within a syncytium reflects the behaviour of multiple cells rather than that of individual iPSC-CMs^26,52,63^. Although not undertaken as part of the present study, it would be of interest to quantify the extent of the effect of electrical coupling on responses to ion channel modulation by making recordings in the presence of gap junction inhibitors. Prior work has shown gap junction inhibition in iPSC-MLs with a ventricular phenotype to depolarise the resting membrane potential by ~ 7.5 ms^27^, demonstrating that direct electrical coupling has profound effects on the electrophysiological phenotype. Recording from iPSC-MLs using IMPASC produced consistent responses to ion channel modulation (Figs. 5, 6, 7). Whilst 17.6% and 86.4% of isolated RA-iPSC-CMs responded to modulation of I_SK_ and I_K,ACh_ respectively, 100% of RA-iPSC-MLs were sensitive to application of UCL1684, apamin and ACh. These data raise the possibility that IMPASC may be of wider value in the study of other ion channels which are inconsistently expressed in isolated iPSC-CMs. For rundown-sensitive currents such as I_Ks_, the extent of coupling (and therefore of intracellular diffusion of pipette solution) may perturb the utility of the IMPASC approach, and therefore further investigations of this will be beneficial in future studies.

### Comparing IMPASC with existing methodologies

Whilst prior studies have recorded *spontaneous* activity in iPSC-MLs through whole-cell patch clamp^27,64–66^ or *triggered* activity using multi-electrode arrays or optical mapping^32,67–71^, the use of whole-cell patch clamp in recording triggered activity in 2D monolayer preparations has previously been limited to iPSC-CMs overexpressing channelrhodopsin (ChR) protein^72–74^. The use of optical mapping is hindered by the generation of data in arbitrary fluorescence units which prevents the quantification of membrane potential. This means such methods are not suitable for assessment of voltage-dependent parameters such as MDP, AP amplitude or plateau amplitude. Experimental characterisation of such voltage-dependent parameters is important in order to allow the translation of data to in silico studies and to allow direct comparisons to be drawn between electrophysiological properties of iPSC-CMs and adult cardiomyocytes. For the study of iPSC-CMs, quantification of voltage, particularly of MDP, is highly beneficial given that membrane potential is commonly used as a key marker of iPSC-CM maturity^16,22,64,75^.

Whilst the rate of fluorescence change in modern dyes used for optical mapping is in the sub-millisecond range, imaging capture is often limited at ≤ 200 frames per second, which is a significant reduction in sampling rate compared with patch clamp^11,71^. This may limit the detection of modest changes in APD, such as those induced by SK channel inhibition in the present study, and also prevents accurate recording of AP upstroke velocity. Optical mapping experiments do however also offer advantages over patch clamp. For example, the ability to measure conduction velocity across a tissue and a significant increase in throughput, and these are particularly beneficial in the use of iPSC-CMs for drug toxicity screening^11,64,76^.

Overexpression of ChR allows 2D monolayers to be optically paced as these light-sensitive channels permit sodium influx in response to exposure to blue light. ChR expression is typically induced by adenoviral infection of iPSC-CMs^72–74^ and subsequently allows triggered whole-cell recordings to be made without generation of stimulation artefacts. Although undoubtedly a useful technique, the need for a light source which can be paced at a given rate and be positioned in close proximity to the iPSC-CM preparation may make it challenging to incorporate into some laboratories.

Electrophysiological recordings of *triggered* APs have also previously been made from multicellular iPSC-CM preparations in the form of EHTs, from which recordings typically involve the use of sharp microelectrodes and for which AP firing is triggered by traditional field stimulation^9,19,21,35^. We found that traditional field stimulation of 2D monolayers produced large stimulation artefacts (Fig. S2), and while these might have been reduced through the use of sharp microelectrodes, this recording method in 2D iPSC-CMs has been reported to be technically very challenging^50^. The culture of iPSC-CMs in EHTs produces isolated cells with a more mature phenotype as characterised by an increase in I_Na_ and I_Ca,L_ in addition to a more hyperpolarised MDP, increased AP amplitude and upregulation of metabolic pathways associated with oxidative phosphorylation^24,35,77,78^. When studied using sharp electrodes in situ, EHTs are reported to exhibit similar diastolic potential and upstroke velocity to left ventricular tissue^21,35^. Generation of EHTs, however, requires a large cell number (typically > 1 × 10^6^/EHT, vs. 2.5 × 10^4^/iPSC-ML in the present study) and culture in specialised systems which may impair the practicality and scalability of such methods for many laboratories^36^. The cell culture methods used in the present study required only changes in plating protocol and alteration of the coverslip material used and therefore represent a highly accessible method to reduce electrophysiological variability. It is plausible to suggest that combining the IMPASC technique presented here with culture techniques shown to increase the maturity of iPSC-CMs^16,79,80^ may address some of the advantages EHTs currently confer over our 2D iPSC-ML system. In particular we note that co-culture of iPSC-CMs with other cell types such as fibroblasts, neurons or endothelial cells has been shown to promote their maturation^81–84^. In the present study, non-cardiomyocytes were removed from the monolayer using lactate enrichment, but the IMPASC technique could easily be employed in the future to investigate the effect of co-culture on iPSC-CM phenotype.

### iPSC-CMs as a model system for the study of SK channel function

Previous investigations of isolated iPSC-CMs (differentiated without RA) showed that application of apamin failed to prolong repolarisation^85,86^, however the effects of SK channel inhibition in RA-iPSC-CMs have not been thoroughly investigated. Differentiation of iPSC-CMs using RA increases transcript levels of *KCNN3* (which encodes the SK subunit isoform SK3) and a trend toward increased *KCNN2* (SK2) transcript levels has also been observed^9^. Despite this, UCL1684 (IC_50_ < 3 nM) was not shown to prolong APD_90_ in RA-iPSC-MLs at concentrations up to 1 µM^71^. Data were collected by optical mapping in the study by Gunawan et al. and sampling rate was limited to 100 frames per second, with which it would be challenging to detect the modest (< 20 ms), but physiologically relevant, APD prolongation recorded in the present study.

Prolongation of repolarisation by apamin or UCL1684 at APD_50_ was significantly larger in RA-iPSC-MLs than in DMSO-iPSC-MLs in the present study (Fig. 7). In the healthy adult heart, apamin and UCL1684 have no effect on ventricular repolarisation and SK channel inhibition selectively prolongs atrial repolarisation^6,37^. Differentiation of iPSC-CMs without RA typically produces a small population of cells which exhibit an atrial-like phenotype, likely originating from a fraction of mesodermal cells which have the inherent capacity to synthesise RA^8,87^. It is possible that this sub-population is responsible for the effects of SK channel inhibition in DMSO-iPSC-MLs, and this is reflected in the fraction of isolated DMSO-iPSC-CMs and DMSO-iPSC-MLs which responded to activation of the atrial-selective current, I_K,ACh_ (Figs. 1 and 6). The prolongation of mid repolarisation recorded following application of apamin or UCL1684 in RA-iPSC-MLs is similar to the reported effects of SK channel inhibition in freshly isolated human CMs or atrial trabeculae^6,39,88^, demonstrating the utility of RA-iPSC-MLs as a model system for the study of I_SK_. Prolongation of APD_90_ by apamin and UCL1684, as occurred in iPSC-MLs which exhibited short, triangular APs (Fig. S6Ai,Bi) also recapitulates the effects I_SK_ inhibition recorded in isolated adult CMs or tissue^6,37,39^.

The abbreviation of APD_90_ by SK channel inhibition however, as recorded in RA-iPSC-MLs which exhibited rapid early repolarisation (Fig. S6Aii,Bii), has not previously been associated with inhibition of I_SK_^6,37,39,88^. We found that abbreviation of repolarisation by increasing the firing rate or by prior application of ACh led to prolongation of APD_90_ when UCL1684 was applied (data not shown), suggesting that this abbreviation was not a *direct* effect of I_SK_ inhibition. UCL1684 caused significant elevation of the plateau phase in this group of RA-iPSC-MLs. Similar elevation (by I_Kur_ inhibition) has previously been shown to increase the contribution of I_Kr_ to repolarisation and therefore to abbreviate APD_90_^89,90^. Given the over-reliance of iPSC-MLs on I_Kr_ for repolarisation demonstrated in Fig. 5, it is plausible to suggest that recruitment of I_Kr_ may be responsible for the abbreviation of repolarisation caused by application of SK channel inhibitors. The over-reliance of iPSC-CMs on I_Kr_ for repolarisation is a feature of their immature electrophysiological phenotype^17,35^. As discussed above, these data suggest that combining IMPASC with methods that improve the maturation state of iPSC-CMs, and subsequently reduce the dependence of iPSC-MLs on I_Kr_ for repolarisation, may be beneficial in the development of an improved in vitro model of cardiac function.

## Methods

### Culture and differentiation of iPSC-CMs

#### Differentiation of induced pluripotent stem cell-derived cardiomyocytes

The REBL-PAT cell line of human iPSCs were a gift from Professor Chris Denning^91^. REBL-PATs were derived from a skin punch biopsy of a healthy male subject. Cells were dedifferentiated into induced pluripotent stem cells using the CytoTune-iPS Sendai Reprogramming Kit^91^ (Thermo Fisher, A16517) and stored in liquid nitrogen until use. Cells were thawed in a water bath at 37 °C then resuspended in Essential 8 complete media (E8; Gibco, A1517001) supplemented with 10 µM Y-27632 and plated in a T25 flask coated with 8.7 μg/cm^2^ growth factor reduced Matrigel (Corning, 734-0268). Media was replenished with E8 media daily until REBL-PATs reached ~ 80% confluency, at which point iPSCs were passaged using Accutase. Once a sufficient yield of iPSCs was produced, cells were plated into 24-well plates for differentiation.

Differentiation was initiated once cells reached 80–90% confluency using a ‘preconditioning’ medium consisting of 500 µl StemPro-34 complete medium (Gibco, 11580356) supplemented with 1 ng/ml BMP4 and Matrigel diluted 1:100^91^. 12–16 h later (day 0) medium was changed to 500 µl StemPro-34 complete medium supplemented with 8 ng/ml Activin A and 10 ng/ml BMP4. At day 2, medium was replaced with 500 μl RPMI1640 (Gibco, 21875034) supplemented with 2% B27^-ins^ (Gibco, A1895602), 10 μM KY02111 and 10 μM XAV939 per well. 1 μM retinoic acid (RA) or a DMSO control was also included in this medium to direct differentiation towards an atrial-like lineage^7,10^. Medium was changed to 500 μl RPMI1640 with 2% B27 supplement (RPMI/B27; Gibco, 11530536) plus 10 μM KY02111 and 10 μM XAV939 with RA or DMSO on day 4, and then to 1 ml RPMI/B27 only on day 6. Subsequently, RPMI/B27 was replenished every 2 or 3 days with 1 or 1.5 ml RPMI/B27 respectively. All solutions were brought to room temperature before use. Media were then made by combining compounds and subsequently sterile-filtered using a 0.2 μm polyethersulfone filter. This protocol is summarised in Fig. S7.

#### Dissociation and freezing of iPSC-CMs

On day 13, iPSC-CMs were dissociated into single cells and frozen for future use^36^. Monolayers were washed in HBSS (Gibco, 14175095) and then incubated for 3–3.5 h at 37 °C in 300 μl HBSS with 1 mM HEPES, 10 μM Y-27632, 30 μM N-Benzyl-p-toluenesulfonamide and 200 U/ml collagenase type II. Subsequently, cells were dissociated by gentle rocking of the plate and diluted with 300 µl RPMI supplemented with 24 μg/ml deoxyribonuclease before centrifugation. iPSC-CMs were resuspended in a seeding media (SM) consisting of RPMI/B27 with 10% FBS and 10 μM Y-27632, triturated into single cells, counted and re-centrifuged. Finally, iPSC-CMs were resuspended in FBS with 10% DMSO and 10 µM Y-27632, transferred to cryovials, and frozen at -80°C in a ‘Mr Frosty’ freezing container (Nalgene) filled with isopropanol. After freezing, iPSC-CMs were transferred to liquid nitrogen for long term storage. The protocol to this timepoint represents the creation of one ‘differentiation batch’ of iPSC-CMs.

#### Thawing and culture of iPSC-CMs

Cryovials of day 13 iPSC-CMs were thawed in a water bath at 37 °C and then diluted slowly with 9 ml SM. Following centrifugation, iPSC-CMs were resuspended in SM and plated into Matrigel-coated (8.7 μg/cm^2^) 24-well plates at ~ 3 × 10^5^/1.9 cm^2^. The following day, media was replaced with 1 ml RPMI/B27. Once monolayers restarted beating (typically by day 15), they were incubated in 1.5 ml RPMI1640 without glucose (Gibco, 11879020) but supplemented with 4 mM sodium-l-lactate. This was replenished after three days and returned to RPMI/B27 after six days. Restriction of glucose and lactate enrichment in this manner works to purify the population of cells into cardiomyocytes only^92^. Subsequently, iPSC-CMs were cultured as a monolayer in 1 or 1.5 ml RPMI/B27 and this was replenished every 2 or 3 days respectively until replating.

#### Replating and culture of isolated iPSC-CMs

iPSC-CMs were cultured as monolayers and then dissociated into single cells using the method outlined above. Single cells, resuspended in SM, were then plated onto glass coverslips coated with 0.1% gelatin. The day after dissociation, SM was replaced with RPMI/B27, which was subsequently replenished every 3–4 days until experimentation. Dissociation was typically performed at day 58–60 and experiments conducted between day 60 and day 70 (termed isolated D65 in Fig. 3). This timeline was followed for collection of all data presented in Figs. 1 and 2, but an earlier timepoint (D35) was also used for the data presented in Fig. 3. For these data, iPSC-CMs were dissociated at day 28–30 and experiments performed between day 30 and 40 (isolated D35).

#### Replating and culture of iPSC-MLs

Following lactate enrichment, iPSC-CMs were cultured in RPMI/B27 until day 27, at which point they were dissociated using the methods outlined above. Cells were resuspended in SM at 2.5 × 10^4^/10 µl. During the dissociation, Matrigel was diluted to 128 µg/ml in ice-cold DMEM and a 10 µl ‘Matrigel spot’ applied to the centre of Thermanox coverslips (Nunc, 174950) in a 24-well plate. This was allowed to polymerise for at least 30 min in a 37 °C cell culture incubator. Matrigel was then removed from the coverslips and spots were rinsed with 10 µl PBS. A 10 µl cell suspension was then placed onto the coated section of each coverslip and allowed to adhere for two hours before 500 μl SM was added gently. The following day, medium was replaced with 200 μl RPMI/B27, and this was replenished every 3–4 days. This method was used to generate the data presented in Fig. 3 (RA-iPSC-MLs) plus Figs. 4, 5, 6 and 7.

Thermanox coverslips were used for these experiments as monolayers of iPSC-CMs were found to not adhere to glass coverslips for more than ~ 5 days. Matrigel was allowed to polymerise in a humidity-controlled cell culture incubator to prevent spots from drying out. The concentration of Matrigel and dilution of cells was determined by calculating the area of a ‘Matrigel spot’ and scaling down the volumes used for normal iPSC-CMs culture in a 24-well plate. This method of replating allowed formation of a syncytium of iPSC-CMs containing ~ 25,000 cells.

### Immunocytochemistry

#### Fixation and immunostaining

Isolated iPSC-CMs were dissociated on to glass coverslips coated with 0.1% gelatin and then cultured for a further 2–12 days as described above. Cells were washed with ice-cold PBS supplemented with 1 mM MgCl_2_ and 0.1 mM CaCl_2_ (PBS^+^) then incubated in 4% PFA at room temperature for 20 min. Subsequently, cells were washed with PBS^+^ and permeabilised using 0.1% Triton X-100 (in PBS^+^). iPSC-CMs were blocked with 2.5% normal horse serum for 30 min. Primary antibodies targeting cTnT (mouse; 1:500; Abcam, ab8295), and/or MLC2v (rabbit; 1:500; Abcam, ab79935) were used in conjunction with DyLight anti-rabbit-488 and DyLight anti-mouse-594 secondary antibodies (Vector Laboratories, DK-8818). Cells were incubated with antibodies for one hour, with a wash in PBS^+^ separating the incubations. After washing with PBS^+^, cells were incubated in 1.2 μM DAPI for 5 min. Finally, coverslips were washed with PBS^+^, then briefly in H_2_O, and mounted to microscope slides using ProLong Gold Antifade Mountant (Thermo Fisher; P10144). All steps were performed at room temperature on a rocking platform and slides were transferred to 4 °C once set.

#### Cellular imaging and analysis

Cells were imaged using a 63 × oil immersion lens on a Leica SP5II confocal microscope. Fluorophores were excited at 405 nm (DAPI), 488 nm (green) or 568 nm (red) using an argon, diode-pumped solid-state or helium–neon laser respectively. Emission was detected from a single confocal plane with 1 nm depth, using sequential scanning at wavelengths of 415–478 nm (DAPI), 498–551 nm (green) and 571–650 nm (red). All images were processed and analysed using FIJI^93^. The percentage of cells positive for proteins was calculated through manual counting of a minimum of three images from each differentiation batch. The percentage of iPSC-CMs that were positive in each batch was calculated and used to determine the mean for each treatment group.

### Electrophysiology

#### Solutions used for electrophysiological recordings

For all recordings, an external solution was used which consisted of (in mM): NaCl (138), KCl (4), HEPES (10), D-glucose (10), MgCl_2_ (1.2), CaCl_2_ (2.5); and titrated to pH 7.4 with NaOH. For recording of action potentials using whole-cell current clamp, and of ACh-sensitive currents using whole-cell voltage clamp, an internal solution was used which consisted of (in mM): KCl (110), NaCl (10), HEPES (10), glucose (5), MgCl_2_ (0.4), K_2_ATP (5) and Tris-GTP (0.5); and titrated to pH 7.1 with KOH (Internal 1). For whole-cell voltage clamp recording of SK-mediated currents in isolated cells, an internal solution was used which consisted of (in mM): K-aspartate (97), KCl (20), Na_2_-ATP (1.5), HEPES-Na (10), EGTA (10), CaCl_2_ (7.68) and MgCl_2_ (1.86); and triturated to pH 7.2 with KOH (Internal 2). Free calcium and magnesium concentrations were calculated to be 500 nM and 500 μM respectively using Max Chelator v8^94^. Internal solutions were aliquoted and stored at − 20 °C. Liquid junction potentials of 5.4 mV (Internal 1) and 9.9 mV (Internal 2) were calculated using the liquid junction potential calculator in Clampex 11.2 (Molecular Devices) but not corrected for.

#### Data acquisition from isolated iPSC-CMs and iPSC-MLs

Glass (isolated cells) or Thermanox (iPSC-MLs) coverslips were placed into a perfusion chamber mounted on an inverted microscope (Zeiss Axiovert S100). Cells were continuously perfused with external solution at room temperature (for single cell current recordings, Figs. 1 and 2) or 37 °C (for AP recordings; Figs. 3, 4, 5, 6 and 7). Patch pipettes were pulled from filamented borosilicate capillaries with an outer diameter of 1.5 mm and an inner diameter of 0.84 mm. Pipette resistances of 2–4 MΩ were used for all experiments and series resistance of up to 10 MΩ were compensated for by at least 70% during voltage clamp recordings. All protocols were designed and applied using Pulse v8.8 (HEKA). For recording of I_K,ACh_ in isolated iPSC-CMs, cells were held at − 40 mV then stepped to + 20 mV for 100 ms before a 250 ms descending ramp was applied to − 120 mV (see inset of Fig. 1Ci,Di). For recording of I_SK_ in isolated cells, iPSC-CMs were held at -40 mV before a 1 s ramp protocol was utilised which ran from − 80 to + 80 mV (see inset of Fig. 2Ai,Bi). iPSC-CMs/MLs were dialysed for at least 2 min before recordings were made. I_K,ACh_ amplitude was measured at -120 mV. I_SK_ amplitude was measured at − 20 mV. Recordings were made using an Axopatch 200B amplifier (Axon instruments) with CV 203BU headstage. Currents were filtered at 1 kHz using a Model 900 filter (eight-pole low-pass Bessel filter; Frequency Devices Inc.) and recordings were sampled at 10 kHz and digitised using an ITC-18 computer interface (Instrutech Corporation). The number of differentiation batches used for each experiment is detailed in the appropriate figure legends.

For triggering of action potentials in iPSC-MLs, Pulse was configured to deliver a TTL signal to a DS3 isolated constant current stimulator (Digitimer) via an output channel. Both the positive and negative output from the DC stimulator were connected to platinum electrodes. The positive electrode was placed inside a borosilicate patch pipette containing external solution and pulled to a resistance of ~ 1–1.5 MΩ. This was mounted on a PatchStar micromanipulator to allow manoeuvrability. The negative electrode was placed in the perfusion chamber. The stimulator was configured to deliver a 5 ms hyperpolarising pulse. Once an iPSC-ML had been successfully patched, the stimulating electrode was positioned in close proximity to the monolayer and a stimulating current of 100 µA was injected at intervals of 1000 ms. The stimulating current was slowly increased to establish the stimulation threshold, which was typically 200–700 µA. Stimulation thresholds could be easily detected through visual inspection of contraction and recording of triggered APs. iPSC-MLs were paced using the stimulation threshold + 20%.

#### Pharmacology

Aliquots of stock solutions of all drugs were stored at -20°C and diluted 1:1000 in external solution on the day of use. ACh (Sigma-Aldrich) and apamin (Bio-techne) were dissolved in H_2_O. UCL1684 (Bio-techne) and E-4031 (Caymen chemicals) were dissolved in DMSO. 100 nM apamin, 100 nM UCL1684 and 1 µM E-4031 was used to maximally inhibit I_SK_ and I_Kr_ respectively^95,96^. 1 µM ACh was used to fully activate I_K,ACh_^97^.

#### Data analysis

All statistical comparisons were made using Prism v9 (GraphPad). Data were analysed using Clampfit v11.2 (Molecular Devices) and a custom written script run using MATLAB R2022a (MathWorks; available upon request). Variability in APD_90_ (see Fig. 3Civ) was calculated as the mean absolute difference in APD_90_ between each AP and its preceding AP for all APs within the recording period. P values of less than 0.05, 0.01, 0.001 and 0.0001 are signified by *, **, *** and **** respectively. Data were tested for normality using the D’Agostino-Pearson omnibus normality test and parametric or non-parametric statistical analyses were performed as appropriate. In all column scatter plots and paired line plots, the different shades represent the independent differentiation batches used. Batches were pooled for comparisons between RA and DMSO. Care has been taken to ensure that one colour is consistently matched to the same batch throughout all figures. The tests used for each comparison are detailed within the legend of the appropriate figures.

### Supplementary Information

Supplementary Information.

## Data Availability

All data generated in the present study are available from the corresponding author upon request.
