# Supplementary material for: In situ monolayer patch clamp of acutely stimulated human iPSC-derived cardiomyocytes promotes consistent electrophysiological responses to SK channel inhibition
Source: Sci Rep. 2024 Feb 7;14:3185. doi: 10.1038/s41598-024-53571-6 (PMC10850090; doi:10.1038/s41598-024-53571-6)
Supplement: Supplementary file 1 — Supplementary Information. [file 41598_2024_53571_MOESM1_ESM.pdf]

Supplementary information for

In situ monolayer patch clamp of acutely stimulated human iPSC-derived cardiomyocytes promotes consistent electrophysiological responses to SK channel inhibition

By

Andrew S. Butler[1], Raimondo Ascione[2], Neil V. Marrion[1], Stephen C. Harmer[1]*, Jules C. Hancox[1]*

[1] School of Physiology, Pharmacology and Neuroscience, University of Bristol, BS8 1TD, United Kingdom.

[2] Bristol Heart Institute and Translational Biomedical Research Centre, Faculty of Health Science, University of Bristol, Bristol BS2 8HW, United Kingdom.

*Authors for correspondence

s.c.harmer@bristol.ac.uk

jules.hancox@bristol.ac.uk

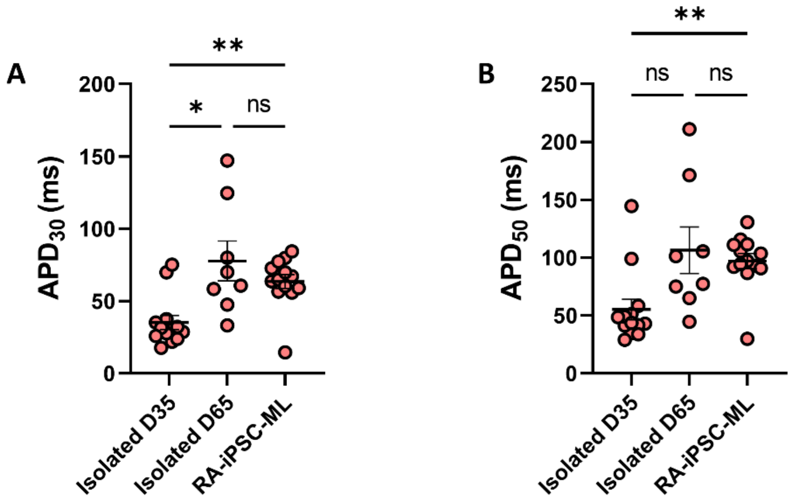

Figure S1: APD_{30} and APD_{50} in isolated RA-iPSC-CMs and RA-iPSC-MLs.
APD_{30} (A) and APD_{50} (B) recorded from isolated D35 RA-iPSC-CMs, isolated D65 RA-iPSC-CMs and RA-iPSC-MLs. All action potentials recorded using whole-cell current clamp at 37 °C. Data presented as mean ± SEM. Statistics represent Brown-Forsythe ANOVA test (as appropriate). Data recorded from one batch of RA-iPSC-CMs. These data are calculated from the same recordings as presented in Fig. 3.

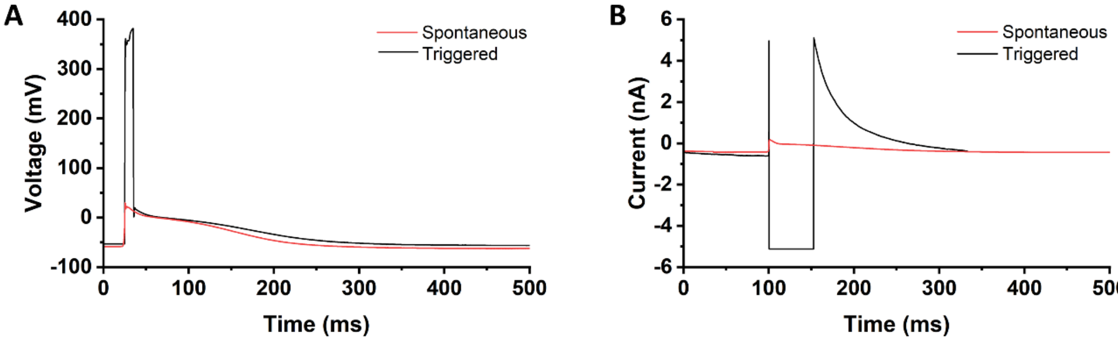

Figure S2: Stimulation of iPSC-MLs.
A) Whole-cell current clamp recording of a spontaneous (red) and triggered (black) APs from an RA-iPSC-ML. AP firing was triggered by using a current injection via the patch electrode. B) Capacitive current recording of a spontaneous (red) and triggered (black) AP from an RA-iPSC-ML. AP firing was triggered by field stimulation with a platinum electrode placed at both edges of the bath. For all recordings, stimulation threshold was confirmed by visual check of the monolayer for contraction and a stimulation of threshold +20% was applied.

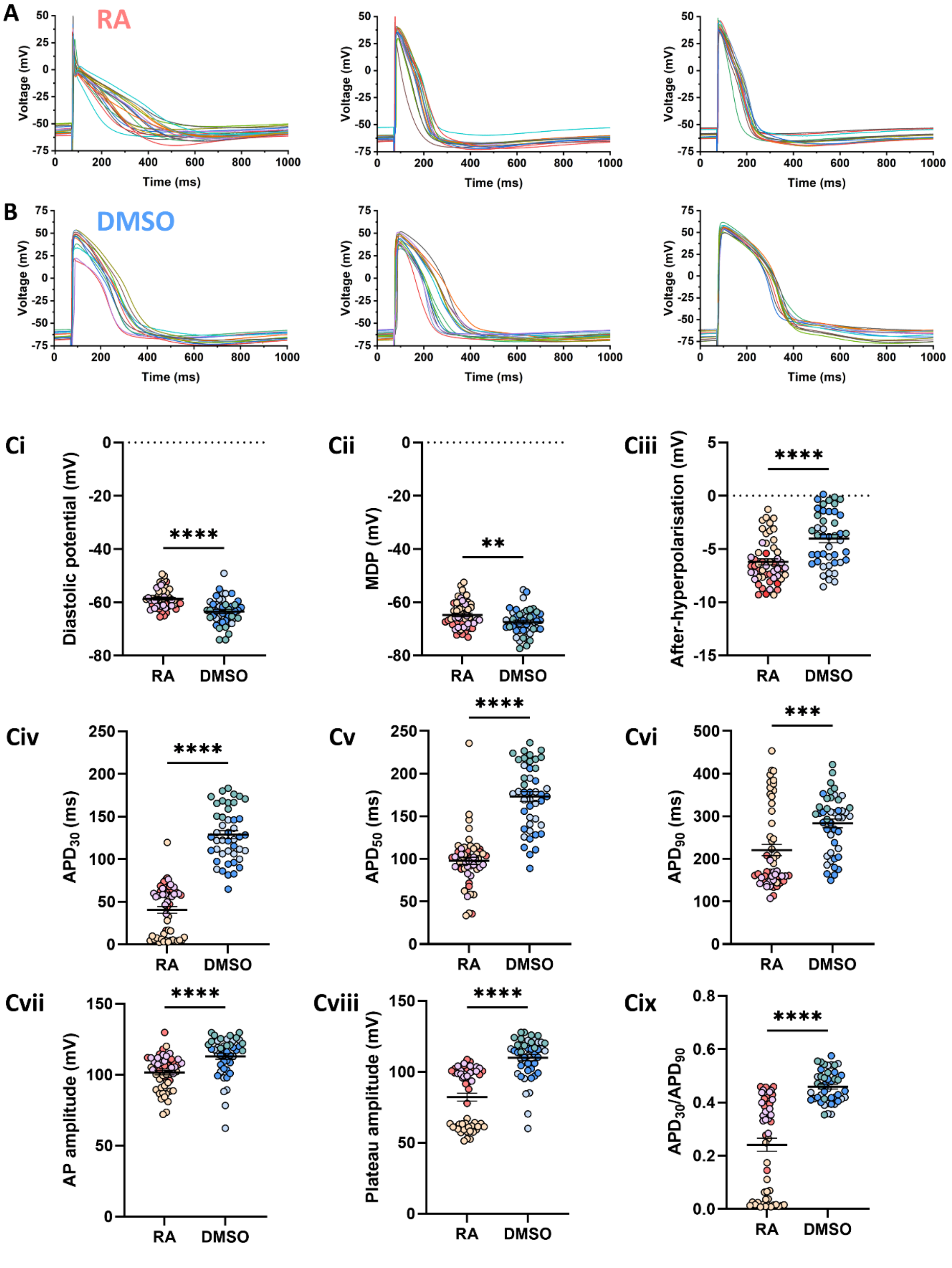

Figure S3: Triggered action potentials in iPSC-MLs.
All action potentials from RA-iPSC-MLs (A) and DMSO-iPSC-MLs (B) used to generate the data presented in Table 1 and Fig. S3C. Traces shown in the three panels represent the three individual differentiation batches of iPSC-MLs used for experiments. In A, *n* = 23, 15 & 15 from left to right. In B, *n* = 15, 17 & 13 from left to right. C) Comparison of properties between RA-iPSC-MLs (reds; *n* = 53) and DMSO-iPSC-MLs (blues; *n* = 45). Diastolic potential (i; membrane potential prior to AP firing), maximum diastolic potential (ii), after-hyperpolarisation (iii; difference between the diastolic and maximum diastolic potential), APD_{30} (iv), APD_{50} (v), APD_{90} (vi), AP amplitude (vii), plateau amplitude (viii; AP amplitude 20 ms after the AP peak) and plateau fraction (ix) are shown. Different shades represent the three differentiation batches. Batches were pooled for comparisons between RA and DMSO. Data presented as mean ± SEM. Statistics represent unpaired Student's t-test, with Welch's correction where appropriate (i, v, viii, ix). APs were recorded using whole-cell current clamp at 37 °C.

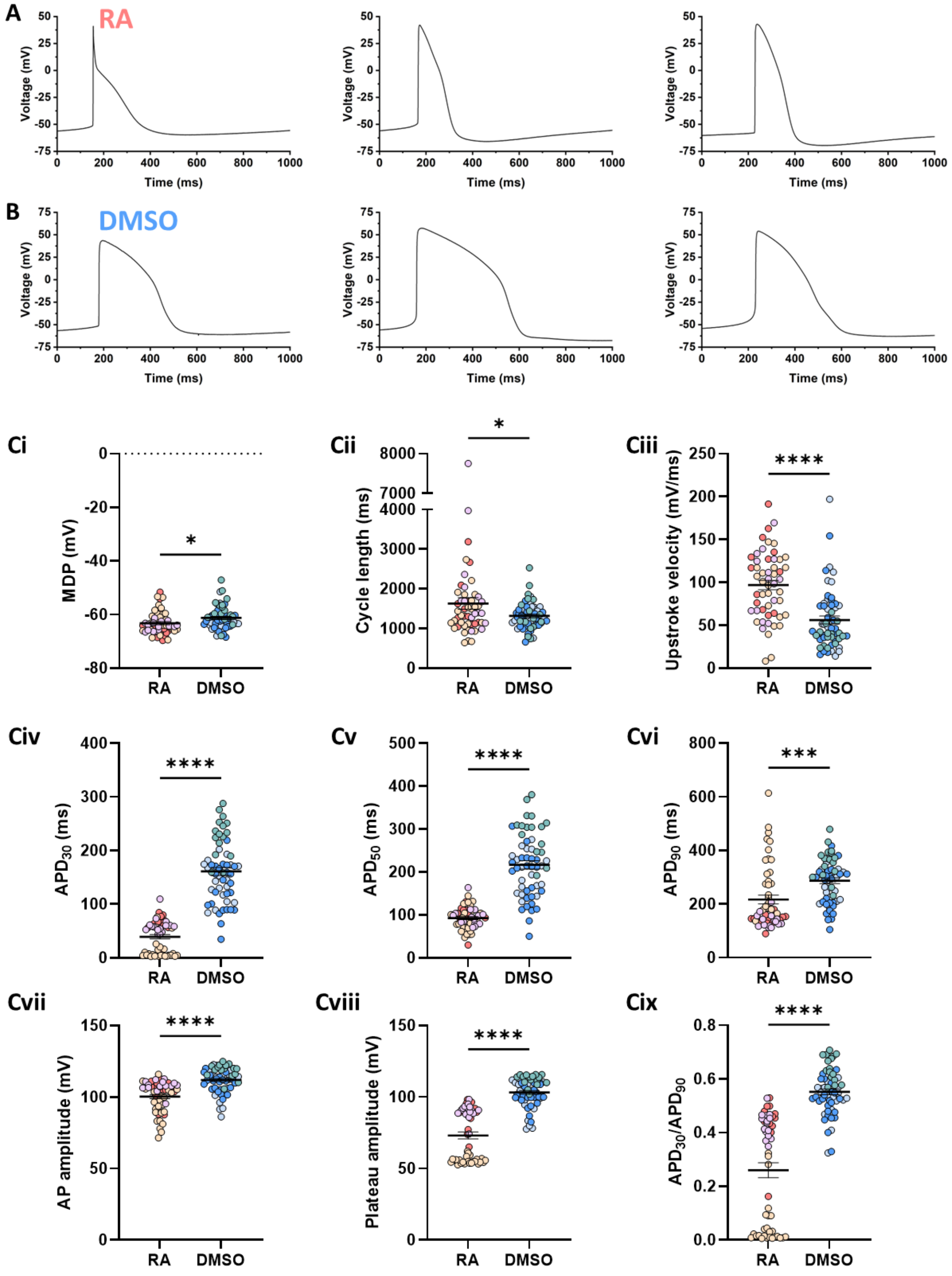

Figure S4: Spontaneous action potentials in iPSC-MLs.
Representative spontaneous action potentials from RA-iPSC-MLs (A) and DMSO-iPSC-MLs (B) used to generate the data presented in Table S1 and Fig. S4C. Traces shown in the three panels represent the three individual differentiation batches of iPSC-MLs used for experiments. C) Comparison of properties between RA-iPSC-MLs (reds; *n* = 53) and DMSO-iPSC-MLs (blues; *n* = 58). Maximum diastolic potential (i), cycle length (ii), maximal upstroke velocity (iii), APD_{30} (iv), APD_{50} (v), APD_{90} (vi), AP amplitude (vii), plateau amplitude (viii) and plateau fraction (ix) are shown. Different shades represent the three differentiation batches. Batches were pooled for comparisons between RA and DMSO. Statistics represent unpaired Student's t-test, with Welch's correction where appropriate (ii, iv, vi, viii & ix). APs were recorded using whole-cell current clamp at 37 °C.

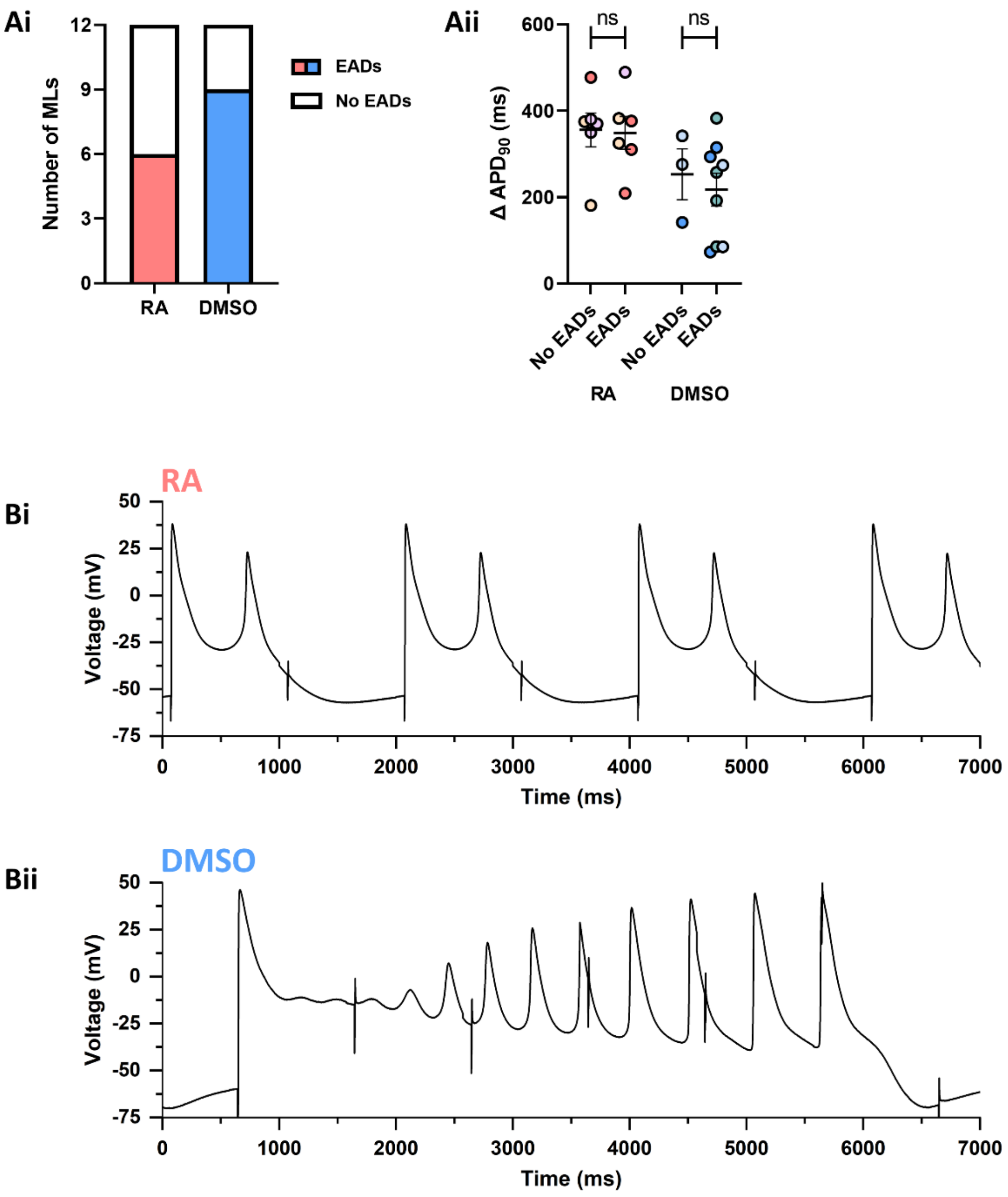

Figure S5: EADs triggered by application of 1 µM E-4031.

Ai) Number of iPSC-MLs in which EADs occurred following application of E-4031. Aii) E-4031-induced prolongation of APD_{90} in RA-iPSC-MLs and DMSO-iPSC-MLs, separated by the occurrence of EADs. Incidence of EADs did not appear to be associated with the degree of APD prolongation. Data presented as mean ± SEM. Statistics represent an unpaired Student's t-test comparing change in APD_{90} between monolayers in which EADs did and did not occur. B) Representative traces from RA-iPSC-MLs (i) and DMSO-iPSC-MLs (ii) exhibiting EADs in the presence of 1 µM E-4031.

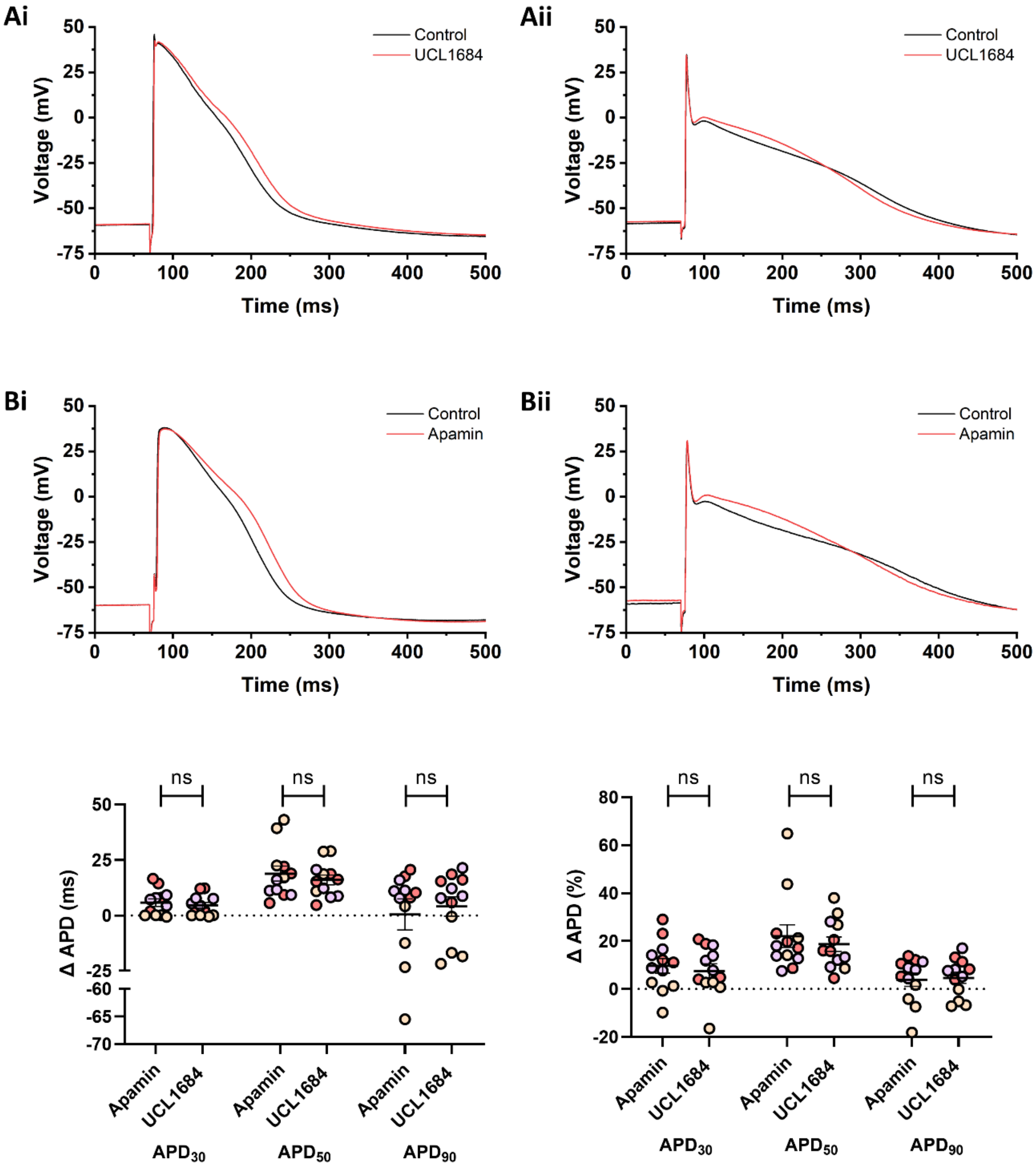

Figure S6: SK channel inhibition abbreviates APD_{90} in RA-iPSC-MLs with rapid phase one repolarisation but not those with a short, triangular morphology.

A) Representative RA-iPSC-CMs with a short triangular AP morphology (i) or with rapid phase one repolarisation (ii) in the absence (black) and presence (red) of 100 nM UCL1684. B) Representative RA-iPSC-CMs with a short triangular AP morphology (i) or with rapid phase 1 repolarisation (ii) in the absence (black) and presence (red) of 100 nM apamin. C) Comparison of the effects of apamin and UCL1684. Data presented as mean ± SEM. Statistics represent an unpaired Student's t test. RA-iPSC-MLs with rapid phase one repolarisation are indicated by the orange circles. *n* = 12 for both groups, consisting of 4 from each differentiation batch. All recordings made at 37 °C.

Day	-12 hours	0 1	2 3	4 5	6 7 8 9 10 11 12	13
Media	**StemPro complete**		**RPMI/B27$^{-insulin}$**	**RPMI/B27**		**Freeze cells**
Supplements	BMP4 (1 ng/ml)	BMP4 (10 ng/ml)	KY02111 (10 μM)			
	Matrigel (1:100)	Activin A (8 ng/ml)	XAV939 (10 μM)			
			± RA (1 μM)			

Figure S7: Culture protocol for the differentiation of iPSCs into cardiomyocytes.
iPSCs were 'preconditioned' ~12 hours before the main differentiation protocol began. In order to push monolayers towards an atrial-like phenotype, retinoic acid (RA) was included in a proportion of monolayers from day 2 to 6. DMSO was used as a control in the absence of RA. Spontaneous beating typically began between day 8 and 10.

Property	RA (n = 53)	DMSO (n = 58)	Significance
**MDP (mV)**	-63.2 ± 0.6	-61.3 ± 0.6	$p < 0.05$
**Cycle length (ms)**	1629 ± 145	1317 ± 43	$p < 0.05$
**Maximal upstroke velocity (mV/ms)**	96.9 ± 5.4	56.2 ± 4.6	$p < 0.0001$
**APD_{30} (ms)**	39.2 ± 4.1	161.1 ± 7.6	$p < 0.0001$
**APD_{50} (ms)**	92.4 ± 3.5	216.7 ± 9.6	$p < 0.0001$
**APD_{90} (ms)**	216.9 ± 16.2	287.2 ± 10.7	$p < 0.001$
**AP amplitude (mV)**	100.3 ± 1.5	111.9 ± 1.1	$p < 0.0001$
**Plateau amplitude (mV)**	73.1 ± 2.4	103.2 ± 1.3	$p < 0.0001$
**Plateau fraction (APD_{30}/APD_{90})**	0.260 ± 0.028	0.553 ± 0.011	$p < 0.0001$

Table S1: Comparison of properties recorded from spontaneous AP in RA-iPSC-MLs and DMSO-iPSC-MLs. Statistics represent unpaired Student's t-test, with Welch's correction where appropriate. Data presented as mean ± SEM. All recordings made at 37 °C. Data pooled from three independent differentiation batches of RA-iPSC-MLs and DMSO-iPSC-MLs. Individual data points can be seen in Fig S4C. Note that upstroke velocity is presented here but not within Table 1 as the stimulation artefact prevented accurate calculations of upstroke velocity from being made in some iPSC-MLs.
